# Are differences in xylem vessel traits and their geographical variation among liana species related to the distribution patterns of climbing mechanisms in a temperate zone?

**DOI:** 10.1093/aob/mcaf138

**Published:** 2025-07-10

**Authors:** Gen Kusakabe, Satoshi Nakaba, Ryo Funada, Tsutom Hiura

**Affiliations:** Department of Ecosystem Studies, Graduate School of Agricultural and Life Sciences, The University of Tokyo, 1-1-1, Yayoi, Bunkyo-ku, Tokyo 113-8657, Japan; Faculty of Agriculture, Tokyo University of Agriculture and Technology, 3-5-8 Saiwai-cho, Fuchu, Tokyo 183-8509, Japan; Institute of Global Innovation Research, Tokyo University of Agriculture and Technology, Fuchu, Tokyo 183-8538, Japan; Faculty of Agriculture, Tokyo University of Agriculture and Technology, 3-5-8 Saiwai-cho, Fuchu, Tokyo 183-8509, Japan; Institute of Global Innovation Research, Tokyo University of Agriculture and Technology, Fuchu, Tokyo 183-8538, Japan; Department of Ecosystem Studies, Graduate School of Agricultural and Life Sciences, The University of Tokyo, 1-1-1, Yayoi, Bunkyo-ku, Tokyo 113-8657, Japan

**Keywords:** functional trait, hydraulic conductivity, Japanese archipelago, root climber, twining climber, vessel dimorphism, woody vine

## Abstract

**Background and Aims:**

Xylem and vessel structures help to maintain water transport in woody plants in response to environmental changes. Many studies have demonstrated the relationships between vessel structures and, in particular, temperature. However, the effects of environmental factors on the vessel size distribution and on vessels of different sizes have not been fully assessed statistically. Lianas are characterized by large vessels and vessel dimorphism. Liana abundance decreases with decreasing temperature; this pattern is attributed to the vulnerability of their large vessels to freeze–thaw embolism. However, in temperate zones, the relationships between liana abundance and temperature differ between twining and root climbers.

**Methods:**

We sampled wood discs of eight liana species distributed across Japan and measured the size and shape of 130 940 vessels in 836 sections from 219 individuals. We classified vessels of each species into two diameter clusters (large and small) and calculated vessel traits and potential hydraulic conductivity (*K*_p_). Vessel traits were compared among climbing mechanisms, and the relationships between vessel traits and temperature were analysed for species.

**Key Results:**

Twining climbers had larger vessel diameters than root climbers and had greater *K*_p_. However, the relationships between temperature and vessel traits of species were inconsistent within climbing mechanisms. The decrease in *K*_p_ in certain species with decreasing temperature might result from species-specific changes in xylem structure. Vessels of the two clusters related differently to temperature in some species.

**Conclusions:**

The vessel traits in each climbing mechanism might partly explain the distribution patterns of these lianas in the study region. Furthermore, changes in *K*_p_ in some species supported the prediction that liana competitiveness decreases with decreasing temperature. Understanding the mechanisms behind the changes in vessel traits and vessel size distribution along environmental factors will provide fundamental insights into how environmental changes affect forest ecosystems by altering plant hydraulic function.

## INTRODUCTION

Maintenance of overall function in woody plants depends on water transport from roots to stem tips, mainly via vessels or tracheids, or both. Given that the water transport capacity of trees is related to their photosynthetic productivity and transpiration (McDowell *et al*., 2019), clarification of the mechanisms behind variations in the traits and functions of water transport pathways (i.e. vessels and tracheids) is essential for understanding individual performance (e.g. photosynthetic rate and growth rate) and ecosystem functions, such as water and carbon fluxes between the forest and atmosphere (Anderegg *et al*., 2018; Flo *et al*., 2021).

Xylem traits reflect trade-offs among water transport and other functions (e.g. mechanical support and nutrient storage) and between the safety and efficiency of water transport (Sperry *et al*., 2008; Lachenbruch and McCulloh, 2014; Gleason *et al*., 2016; Morris *et al*., 2016; Pratt and Jacobsen, 2017). An increase in the fraction of conductive tissue in the xylem leads to an increase in the water transport capacity (Morris *et al*., 2016; Pratt and Jacobsen, 2017). However, increasing the fraction of conductive tissue can incur costs, such as reductions in wood strength, longevity and the storage of defensive compounds and carbohydrates (Pratt and Jacobsen, 2017). The water transport efficiency of individual conduits is determined primarily by their size, which is typically represented by cell diameter (Isasa *et al*., 2023). Ideally, the flow rate per unit time of the conduit increases with the fourth power of the diameter by following the Hagen–Poiseuille law (Tyree and Zimmermann, 2002). At the same time, an increase in conduit size increases the vulnerability of the plant to loss of water transport function in stressful conditions, such as drought, physical damage and, in particular, freezing (Davis *et al*., 1999; Venturas *et al*., 2017; Isasa *et al*., 2023).

Environmental factors influence vessel traits, although path length remains the main determinant of the mean conduit diameter in transverse sections of the stem (Savage *et al*., 2010; Olson *et al*., 2018, 2020). Notably, temperature has been shown to be positively correlated with vessel diameter in natural conditions, even when path length is accounted for (Olson *et al*., 2018, 2020). This correlation has been attributed to natural selection favouring resistance to freeze–thaw embolism (Davis *et al*., 1999; Schreiber *et al*., 2015; Lobos-Catalán and Jiménez-Castillo, 2023) or to adaptation to increased transpiration demand (Olson *et al*., 2020), or both.

Numerous studies have revealed the relationships between environmental factors and conduit diameter (e.g. Venturas *et al*., 2017; García-Cervigón *et al*., 2018, 2020; Olson *et al*., 2018, 2020; Fajardo *et al*., 2022; Olson, 2022). However, these studies have focused mainly on traits described as representative, such as mean diameter and hydraulically weighted diameter (*D*_h_). There are variations in the size of the conduits arranged in xylem sections, and the size distribution of vessels varies among species and individuals (Fajardo *et al*., 2022). For example, certain ring-porous species, which are common in temperate zones, and lianas exhibit bimodal frequency distributions of conduit diameter; this is known as vessel dimorphism (Carlquist, 1981, 2012; Utsumi *et al*., 1999; Rosell and Olson, 2014; Schreiber *et al*., 2015; Gerolamo and Angyalossy, 2017; Pellissari *et al*., 2018; Chery et al., 2020). Large vessels are likely to facilitate efficient water transport, whereas small vessels might provide safety by maintaining functionality when the large vessels are dysfunctional (Carlquist, 2012; Jiménez-Castillo and Lusk, 2013). If this hypothesis is correct, environmental factors are likely to affect vessels of different sizes in distinct ways. For example, changes in vessel traits in response to freezing or drought stress have been observed predominantly in large vessels (Schreiber *et al*., 2015; Hacke *et al*., 2017). However, few studies have examined statistically how environmental factors influence the size distribution of conduits (Medeiros and Pockman, 2014; Schreiber *et al*., 2015; García-Cervigón *et al*., 2018, 2020).

Lianas are woody plants that rely on other structures to support their weight (Schnitzer, 2018), and they influence ecosystem functions such as carbon accumulation (van der Heijden *et al*., 2013, 2015) and transpiration (Ichihashi *et al*., 2017). Lianas are characterized by large vessels (Carlquist, 1985); this has been attributed to the fact that they meet transpiration demands with a relatively small cross-sectional area per unit of leaf mass (Isnard and Feild, 2015; Schnitzer, 2018) or that their vessel size is appropriate to their long stems (Rosell and Olson, 2014, Rosell *et al*., 2017), or both. In addition, lianas are known for exhibiting considerable variation in xylem architecture, including the presence of vessel dimorphism (Rosell and Olson, 2014; Isnard and Feild, 2015; Gerolamo and Angyalossy, 2017; Pellissari *et al*., 2018; Chery et al., 2020; Zhang *et al*., 2023). The species richness and abundance of lianas decrease more rapidly than in other plant growth forms with decreasing temperature (Schnitzer, 2005; Jiménez-Castillo *et al*., 2007; Hu *et al*., 2010). This pattern has been interpreted to indicate that the large vessels of lianas are more vulnerable than the small vessels to freeze–thaw embolism and therefore that lianas decrease the competitiveness with other growth forms of plants in cold environments (Schnitzer, 2005; Jiménez-Castillo *et al*., 2007; Jiménez-Castillo and Lusk, 2013; Lobos-Catalán and Jiménez-Castillo, 2023). However, several studies observed abundant lianas in some temperate and sub-boreal forests where freezing occurs from winter to early spring and reported no relationships between liana abundance and mean annual temperature (MAT) (Lobos-Catalán and Jiménez-Castillo, 2019; Kusakabe *et al*., 2023). Furthermore, in our previous study, the relationship between liana abundance and MAT across a sub-tropical to sub-boreal region varied between twining climbers and root climbers (Kusakabe *et al*., 2023). The former climb by twining their stems, and the latter climb along the trunk of the host by using adventitious adhesive roots. The stem density (number of liana stems per unit area) was positively related to MAT in twining climbers but not in root climbers (Kusakabe *et al*., 2023). Furthermore, inventory data of lianas from multiple sites implicate that the relative abundance of twining climbers and several other climbing mechanisms in the liana community tend to decrease from tropics to temperate regions, whereas that of root climbers tend to increase (Londré and Schnitzer, 2006; Gianoli *et al*., 2010; Castagneri *et al*., 2013; Barik *et al*., 2015; Parthasarathy *et al*., 2015). These results suggest that: (1) resistance to freeze–thaw embolism differs by liana species and climbing mechanism; (2) lianas distributed in cold environments have xylem that is resistant to freeze–thaw embolism; and (3) twining climbers have greater variation in water transport capacity than root climbers along a temperature gradient.

Here, we aimed to elucidate differences in xylem vessel traits among climbing mechanisms and among species, in addition to the variations of these traits in relationship to MAT, in eight liana species distributed in the temperate zone in Japan. We also attempted to evaluate changes in vessel size distribution along a temperature gradient. Given that the xylem structures of lianas exhibit large variation, including vessel dimorphism, lianas appear to be suited for examining the general applicability of methods for evaluating vessels with different size. We expected that this examination would provide a perspective for interpretation of the diversity in traits, functions and distributions among liana species. Although these differences among lianas have been recognized (Darwin, 1865; Isnard and Silk, 2009; Gianoli, 2015; Sperotto *et al*., 2020), they are often considered as a single group of woody plants in ecological studies (but see Sperotto *et al*., 2023; Dias *et al*., 2024). In addition, we anticipated that the results would provide fundamental information on changes in potential water transport, plant distribution and forest ecosystem functions in response to environmental change. We tested the following hypotheses: (1) twining climbers have larger vessel diameters and greater water transport capacity than root climbers; (2) the diameter and density of large vessels and the fraction of all vessels decrease with decreasing temperature, hence the water transport capacity also decreases; and (3) twining climbers have greater changes than root climbers in vessel diameter and water transport capacity along a temperature gradient than root climbers have.

## MATERIALS AND METHODS

### Sampling

**Fig. 1.**
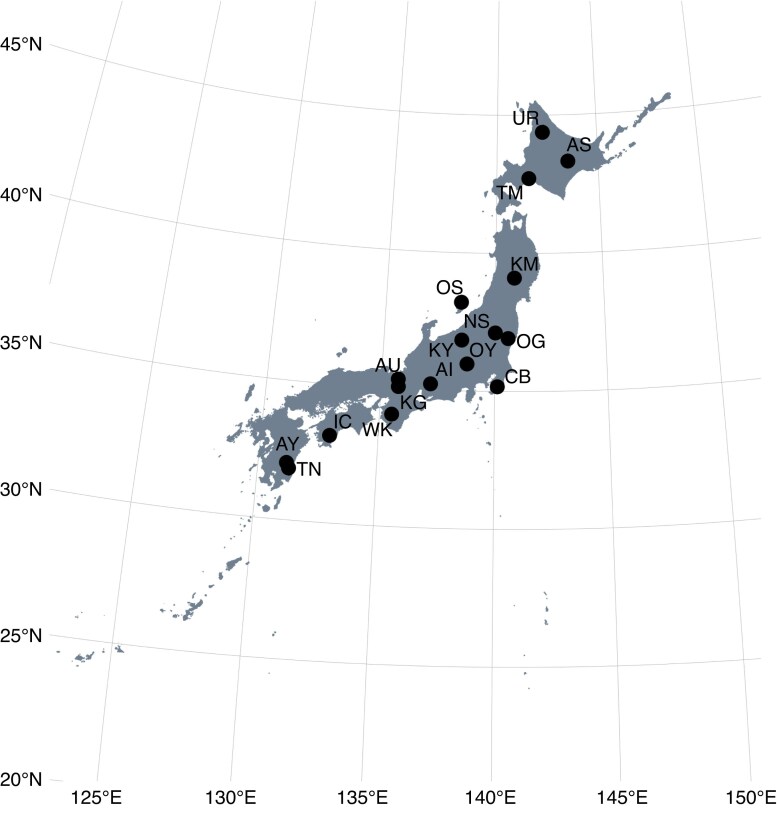
Locations of sampling sites in Japan. Filled circles indicate sampling sites. Site names corresponding to the codes are provided in Table 1. See Supplementary Data Fig. S1 for number of sampling sites for each species.

**Fig. 2.**
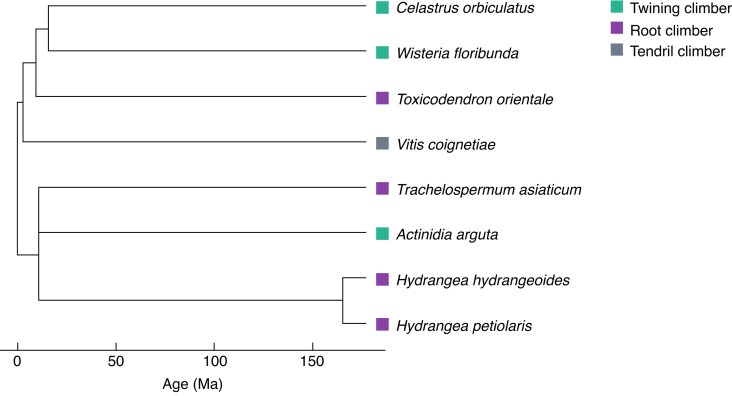
Phylogenetic relationships among the studied liana species. The phylogenetic tree was constructed by extracting data from the large phylogenetic tree in the work of Janssens *et al*. (2020). *Vitis coignetiae* was not included in the dataset; therefore, information on *Vitis vinifera*, which is phylogenetically closely related to *V. coignetiae* (Tröndle *et al*., 2010; Péros *et al*., 2011), was used instead.

Sampling was conducted in July–November 2020 and April–October 2021 at 17 forest sites located in warm temperate and sub-boreal zones of the Japanese archipelago (Fig. 1; Table 1). The study sites are among the core sites of the Monitoring Sites 1000 Project initiated by the Japanese Ministry of the Environment (Ishihara *et al*., 2011), with the exception of the University of Tokyo Chiba Forest (site CB). The sampling points ranged from 31.9°N to 44.4°N. We selected eight liana species that are relatively widespread and abundant in this region (Fig. 2; Table 2). All species are widely distributed across East Aisa, including the Japanese archipelago, and usually reach the canopy layer when they mature. *Actinidia arguta* (Siebold et Zucc.) Planch. ex Miq., *Celastrus orbiculatus* Thunb., *Hydrangea hydrangeoides* (Siebold et Zucc.) B. Schulz, *Hydrangea petiolaris* Siebold et Zucc. and *Toxicodendron orientale* Greene are distributed throughout the sampling area in this study. *Wisteria floribunda* (Willd.) DC. and *Trachelospermum asiaticum* (Siebold et Zucc.) Nakai reach their northern distribution limit in central Japan and southern Tohoku region, respectively. Therefore, these two species are absent from Hokkaido region, where three sampling sites (UR, AS and TM) are located. *Vitis coignetiae* Pulliat ex Planch. are not distributed in Kyushu region, where two sampling sites (AY, TN) are located. All species are members of different orders, with the exception of the two *Hydrangea* species (Table 2). The species comprise three twining climbers, four root climbers and one tendril climber. All species except for *T. asiaticum* (an evergreen broadleaf) are deciduous broadleaf lianas.

**Table 1.** Description of sampling sites.

Site name	Site code	Latitude (°N)	Longitude (°E)	Elevation (m)	Mean annual temperature (°C)
Uryu	UR	44.38	142.27	410	4.52
Ashoro	AS	43.32	143.51	410	5.36
Tomakomai	TM	42.71	141.57	90	7.66
Kanumasawa	KM	39.11	140.87	470	9.24
Osado	OS	38.21	138.44	780	10.15
Nasu	NS	37.12	140.01	960	9.21
Ogawa	OG	36.92	140.59	680	9.6
Kayanodaira	KY	36.84	138.5	1500	5.19
Ohyamazawa	OY	35.98	138.76	1050	8.45
Ashiu	AU	35.34	135.74	770	10.55
Aichiakatsu	AI	35.22	137.17	360	13.22
Chiba	CB	35.17	140.12	280	14.79
Kamigamo	KG	35.07	135.76	140	15.48
Wakayama	WK	34.06	135.53	760	10.69
Ichinomata	IC	33.15	132.92	460	12.74
Aya	AY	32.05	131.19	590	14.12
Tano	TN	31.86	131.3	150	16.91

Each value is the median value for all individuals sampled at the site.

**Table 2.** Description of liana species used in the study.

Species	Order	Family	Climbing mechanism	Leaf phenology	Number of sampling sites	Latitude (°N)	Number of sampled individuals
*Actinidia arguta* (Siebold et Zucc.) Planch. ex Miq.	Ericales	Actinidiaceae	twining	DB	13	31.86–44.39	44
*Celastrus orbiculatus* Thunb.	Celastrales	Celastraceae	twining	DB	9	31.86–43.32	25
*Wisteria floribunda* (Willd.) DC.	Fabales	Fabaceae	twining	DB	6	34.06–39.11	16
*Hydrangea hydrangeoides* (Siebold et Zucc.) B. Schulz	Cornales	Hydrangeaceae	root	DB	13	32.04–44.39	40
*Hydrangea petiolaris* Siebold et Zucc.	Cornales	Hydrangeaceae	root	DB	10	34.05–44.39	31
*Toxicodendron orientale* Greene	Sapindales	Anacardiaceae	root	DB	6	35.17–44.39	18
*Trachelospermum asiaticum* (Siebold et Zucc.) Nakai	Gentianales	Apocynaceae	root	EB	5	31.86–35.23	19
*Vitis coignetiae* Pulliat ex Planch.	Vitales	Vitaceae	tendril	DB	8	35.97–44.37	26

Latitude indicates the lowest-highest values of the location of sampled individual. Abbreviations: DB, deciduous broad leaves; EB, evergreen broad leaves.

In each forest, we chose species for which a sufficient number of individuals were available. We selected three to five individuals of each species with a diameter of 2–4 cm at breast height (∼130 cm height) and a height of ∼10–15 m. Locations of individuals were recorded on a handheld GPS device (GPSMAP 62sc; Garmin International Inc., Olathe, KS, USA). The diameter at breast height and height were measured, and wood discs at breast height were collected. The sample size ranged from 16 to 44 individuals of each species across 5–13 forests (Table 2; Supplementary Data Fig. S1), and 95.7 % (67/70) of the species–site combinations had at least three wood discs. The discs were stored in a solution of 30–35 % ethanol.

### Measurement of vessel traits

**Fig. 3.**
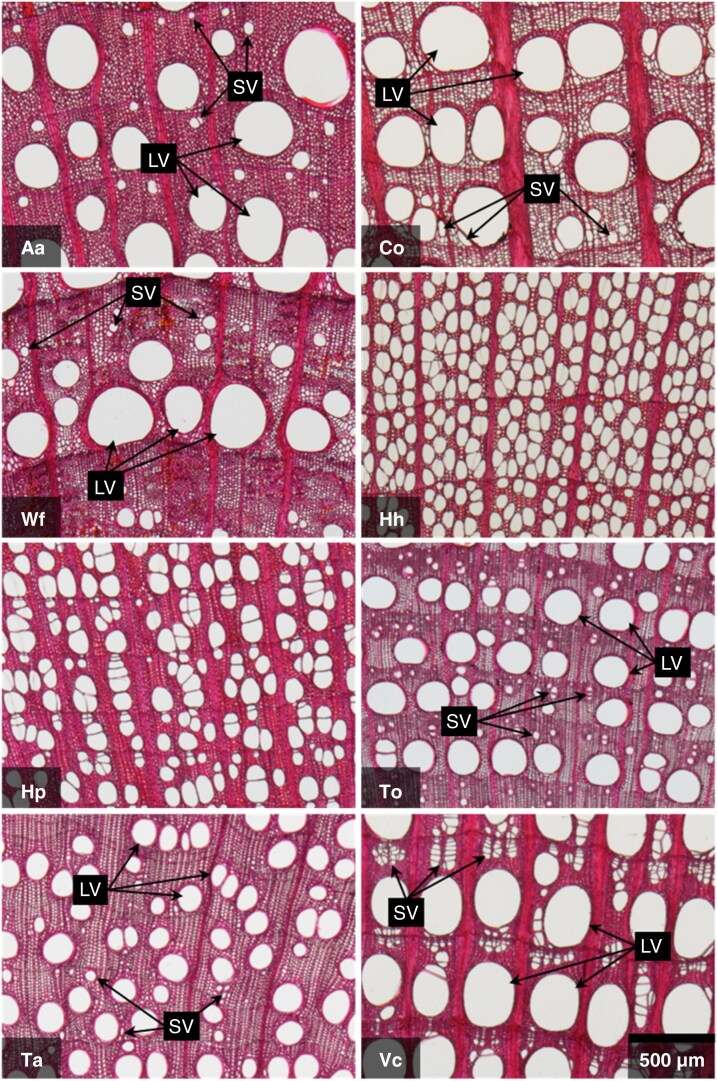
Optical microscopic images of typical xylem transverse sections of the study species. Abbreviations: Aa, *Actinidia arguta*; Co, *Celastrus orbiculatus*; Hh, *Hydrangea hydrangeoides*; Hp, *Hydrangea petiolaris*; LV, large vessels; SV, small vessels; Ta, *Trachelospermum asiaticum*; To, *Toxicodendron orientale*; Vc, *Vitis coignetiae*; Wf, *Wisteria floribunda*. The scale bar in the bottom right of the image of Vc indicates 500 μm. All images are on the same scale.

Wood blocks of 5–10 mm on each side were cut from the discs. All selected species exhibited growth rings in their xylem. Therefore, the wood blocks were obtained to include at least three growth rings from the year before sampling, and ∼30-μm-thick transverse sections were obtained by using a sliding microtome equipped with a freezing device (REM-710; Yamato Kohki, Saitama, Japan) according to Nakaba *et al*. (2015). Blocks and sections were obtained from four different directions on each disc to account for the eccentricity of stems. The sections were stained for at ≥1 min in a 1 % aqueous solution of Safranin. After removal of the excess dye, the sections were dehydrated through an ethanol series (30, 50, 70, 90 and 100 %), after which the ethanol was replaced by xylene. The sections were then embedded in mounting medium (Entellan new; Sigma-Aldrich, Merck, Darmstadt, Hesse, Germany) to create semi-permanent preparations. Images of the sections were captured with a digital camera (DS-Fi2; Nikon, Tokyo, Japan) mounted on an optical microscope (Axio Scope.A1; Carl Zeiss, Oberkochen, Germany) with a ×2.5 objective lens (Fig. 3).

**Fig. 4.**
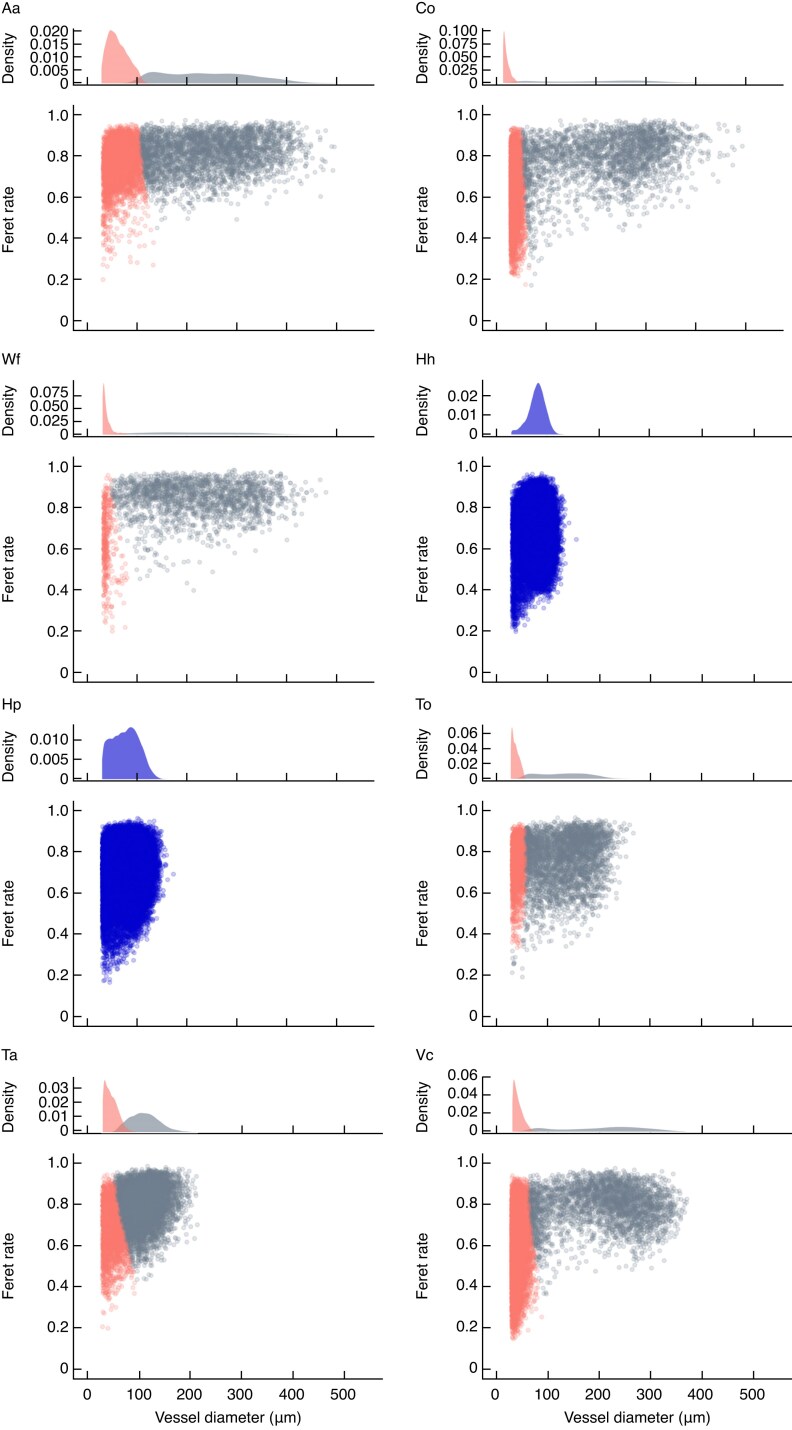
Classification of vessels by Gaussian mixture models using diameter and Feret rate. Each point represents the value for each vessel: red, small-vessel clusters; grey, large-vessel clusters; blue, vessels for species not showing vessel dimorphism. Lower plots in each panel indicate the vessel distribution on the dimension of the diameter and Feret rate (minimum Feret diameter/maximum Feret diameter). Upper plots in each panel indicate the frequency distribution of each vessel cluster. Clustering was not performed for species that did not show vessel dimorphism (*Hydrangea hydrangeoides* and *Hydrangea petiolaris*). Abbreviations: Aa, *Actinidia arguta*; Co, *Celastrus orbiculatus*; Hh, *H. hydrangeoides*; Hp, *H. petiolaris*; Ta, *Trachelospermum asiaticum*; To, *Toxicodendron orientale*; Vc, *Vitis coignetiae*; Wf, *Wisteria floribunda*.

ImageJ-Fiji software (Schindelin *et al*., 2012) was used to detect the measurement area and to measure the images. R v.4.4.1 software (R Core Team, 2024) and the ‘tidyverse’ package (Wickham *et al*., 2019) were used to format and visualize the data. For sections requiring multiple images to cover the entire measurement area, the images were combined by using the ‘MosaicJ’ plugin (Thévenaz and Unser, 2007) of ImageJ-Fiji. In each section, three to five growth rings from the year before the sampling were measured. This measurement criterion was determined to satisfy the following conditions as much as possible: (1) to exclude immature vessels; (2) to capture a sufficient number of vessels; (3) to maintain the relationship between stem diameter and vessel traits to account for plant size in the analyses; and (4) to match years of vessel formation with years of climatic information, used later. Boundaries between the ray parenchyma and other tissues were used to set the boundaries of the radial direction of the measurement area of each image. We excluded from the measurements areas with apparent artificial injury and vessels occluded with substances such as tyloses or gums. As a result, a total of 836 images of sections from 219 individuals were measured. The extracted images were then binarized by using the ‘minimal’ algorithm in ImageJ-Fiji. For binarized images of *H. hydrangeoides*, *H. petiolaris* and *V. coignetiae*, which contained many grouped vessels, a watershed transformation was applied in ImageJ-Fiji. This application separated the hollows that were identified as connected during the binarization process but separated by thin membranes, such as perforation plates and inter-vessel pits. After this procedure, all hollows in the images were measured and the diameters calculated, assuming that the hollows were circular. Subsequently, frequency distributions of the diameters for each individual were drawn, and hollows with diameter ≥ 30 μm were assumed to be vessels in order to minimize the misidentification of non-vessel hollows. A total of 130 940 hollows were identified as vessels. Based on the frequency distribution of vessel diameters and cross-sectional images for each species, we determined that vessel dimorphism was present in *A. arguta*, *C. orbiculatus*, *V. coignetiae*, *W. floribunda*, *T. orientale* and *T. asiaticum* (Fig. 3). For species exhibiting vessel dimorphism, vessels were classified into two clusters (i.e. large-vessel cluster and small-vessel cluster) by using Gaussian mixture models (GMMs) to consider functional differences in the vessels. Lianas with vessel dimorphism often have large vessels that are solitary and circular to oval in shape and small vessels that are chains or clusters and tend to be flattened in shape (Gerolamo and Angyalossy, 2017; Chery et al., 2020). Therefore, the two clusters were fitted as two-dimensional Gaussian distributions with the Feret rate (minimum Feret diameter/maximum Feret diameter; Supplementary Data Fig. S2), which was assumed as a measure of shapes, and vessel diameter as variables (Fig. 4). The two-dimensional Gaussian distributions were aligned parallel to the axis of the variables. The GMMs were applied by using the ‘VVI’ model in the Mclust function of the ‘mclust’ package (Scrucca *et al*., 2016) in R.

We calculated the mean vessel diameter (VD_mean), geometric mean vessel diameter (VD_geomean), vessel density (V_density), vessel fraction (V_fraction) and mean hydraulically weighted vessel diameter (*D*_h_) of each section as vessel traits for the overall vessels and for each cluster. We also extracted the maximum vessel diameter for the overall vessels (VD_max). The V_fraction was calculated as the ratio of total vessel area to the measurement area. The VD_mean and VD_geomean, were calculated according to eqns (1) and (2), respectively, and the *D*_h_ was calculated according to eqn (3) (Tyree and Zimmermann, 1971; 2002; Steppe and Lemeur, 2007):


(1) $$\mathrm{VD}\_\mathrm{mean}=\frac{1}{n}\overset{n}{\underset{i=1}{\sum }}\;d(\mu m)$$

(2) $$\mathrm{VD}\_\mathrm{geomean}={\left(\overset{n}{\underset{i=1}{\prod }}\;d\right)}^{\frac{1}{n}}(\mu m)$$

(3) $$D_{h}={\left[\frac{1}{n}\overset{n}{\underset{i=1}{\sum }}\;d^{4}\right]}^{\frac{1}{4}}(\mu m)$$

where *d* is the diameter of each vessel. Potential hydraulic conductivity (*K*_p_) was calculated by using the following equation (Sterck *et al*., 2008, Poorter *et al*., 2010):


(4) $$K_{p}=\frac{\pi \rho_{w}}{128\eta }\cdot D_{h}^{4}\cdot V\_\mathrm{density}(\mathrm{kg}\;m^{-1}\mathrm{MP}a^{-1}s^{-1})$$

where η is the viscosity of water at 20 °C (1.002 × 10^−3^ Pa s at 20 °C), and ρ_w_ is the density of water at 20 °C (998.2 kg m^−3^).

### Climatic factors

Temperature data were obtained from the Agro-Meteorological Grid Square Data of the National Agriculture and Food Research Organization (Ohno *et al*., 2016). Mean daily temperatures for the 5 years from the year before sampling were obtained from the 1-km-mesh grid cells that included the location of each individual. The MAT was calculated with the data obtained. We expected freeze–thaw to influence the differences in vessel traits. However, because the frequency and intensity of freezing exhibited strong negative correlations with MAT in this region (Supplementary Data Fig. S3), only the relationships of vessel traits with MAT are presented here.

### Statistical analyses

To test the relationships between environmental factors and xylem traits, we should consider the influence of plant size. The mean vessel diameter at a given transverse section scales with the plant size corresponding to the path length from the stem tips to that point (West *et al*., 1999; Savage *et al*., 2010; Rosell *et al*., 2017; Olson *et al*., 2021). Therefore, the mean vessel diameter at a given point of the plant is also scaled with the stem diameter at that point (Savage *et al*., 2010; Olson *et al*., 2013; Olson and Rosell, 2013), although lianas show greater variability than trees in the scaling relationship between xylem traits and stem diameter because of a larger error in the relationship between stem diameter and path length (Rosell and Olson, 2014). In addition, the height of lianas appeared not to correspond to their stem length, owing to their complex shape. Therefore, we used stem diameter to account for the influence of plant size in this study. Before testing the relationships between MAT and vessel traits, we examined the relationships between MAT and the log_10_-transformed values of stem diameter of individuals of each species. *t-*Tests used the Pearson product–moment correlation coefficient as the test statistic. No species showed a significant correlation between the stem diameter of individuals and MAT at the sample location, with the exception of *A. arguta* (*r* = 0.424, *P* = 0.004; Supplementary Data Fig. S4). For *A. arguta*, however, the absolute value of the correlation coefficient was <0.7, hence the collinearity was considered acceptable (Dormann *et al*., 2013). No significant differences in the stem diameter of individuals were found among climbing mechanisms or species (Kruskal–Wallis test, *P* = 0.097 among climbing mechanisms; *P* = 0.259 among species; Supplementary Data Fig. S5). The number of species used in this study was too small to test for phylogenetic signals in vessel traits. Therefore, we did not include phylogenetic information in subsequent analyses.

Differences in vessel traits among climbing mechanisms and among species were modelled by using linear mixed-effects models (LMMs):


(5) $$y_{i}=\beta_{0}+\beta_{1}x_{1}+\gamma_{1i}+\gamma_{2i}+e_{i}$$

where *y*_i_ is the vessel trait value at transverse section *i*, *x*_1_ is climbing mechanism or species, β_0_ is a partial regression constant, β_1_ is a partial regression coefficient determined for each climbing mechanism or species, γ_1i_ and γ_2i_ are random terms for individual and site, respectively, and *e*_i_ is the residual for *y*_i_. Multiple comparisons were conducted by using Tukey's honestly significant difference test for each trait.

The relationships between MAT and xylem vessel traits were tested by LMMs. Traits, except for those described as proportional values (V_fraction of overall vessels and each vessel cluster), were modelled using eqn (6). The V_fraction (proportional values) was logit-transformed in accordance with the methods of Warton and Hui (2011) and modelled using eqn (7):


(6) $$\log_{10}\;(y_{i})=\;\beta_{0}+\beta_{1}x_{1}+\beta_{2}\log_{10}\;(x_{2})+\gamma_{1i}+\gamma_{2i}+e_{i}$$

(7) $$\ln\;\left(\frac{y_{i}\;+\;\varepsilon }{1\;-\;y_{i}\;+\;\varepsilon \;}\right)=\beta_{0}+\beta_{1}x_{1}+\beta_{2}\log_{10}\;(x_{2})+\gamma_{1i}+\gamma_{2i}+e_{i}$$

where *y*_i_ is the vessel trait value at section *i*, *x*_1_ is MAT, *x*_2_ is stem diameter, β_0_ is a partial regression constant, β_1_ and β_2_ are partial regression coefficients for each explanatory variable, ε is the minimum value of *y* and 1 − *y* for each trait, γ_1i_ and γ_2i_ are random terms for individual and site, respectively, and *e*_i_ is the residual for *y*_i_. A Wald test was conducted on the partial regression coefficients of the models.

Statistical analysis was performed in R v.4.4.1 software (R Core Team, 2024). The glmmTMB function in the ‘glmmTMB’ package (Brooks *et al*., 2017) was used to construct LMMs, and the glht function in the ‘multcomp’ package (Hothorn *et al*., 2008) was used for multiple comparisons.

## RESULTS

### Differences in vessel traits among climbing mechanisms and among species

**Fig. 5.**
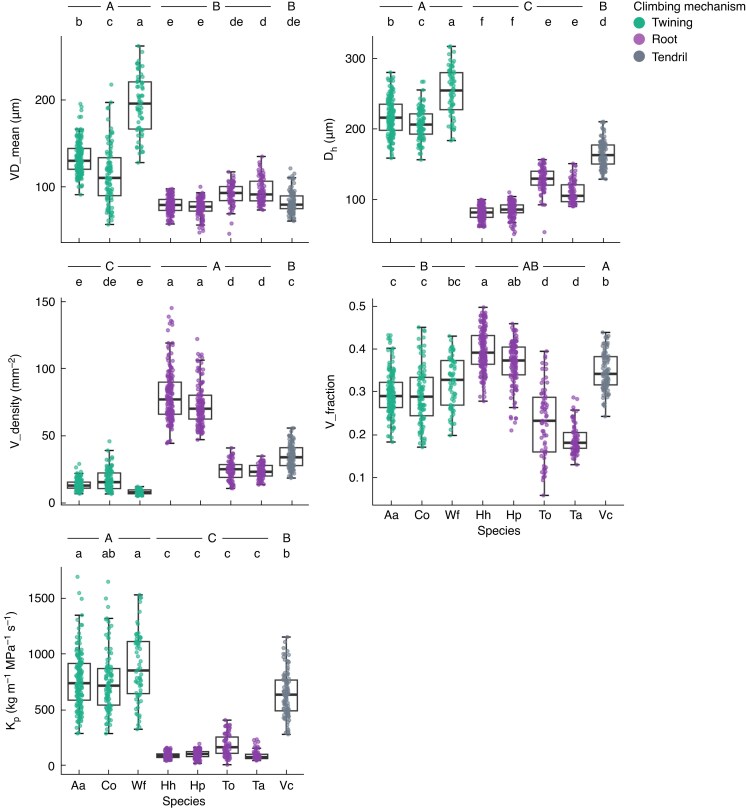
Differences in vessel traits among climbing mechanisms and among species of lianas. Each point represents the value for each transverse section. Letters at the top of each panel are assigned in descending order of mean trait values; different letters indicate significant differences in mean trait values between species or climbing mechanisms (Tukey's honestly significant difference test, *P* < 0.05). Upper-case letters indicate differences between climbing mechanisms, and lower-case letters indicate differences between species. Abbreviations: Aa, *Actinidia arguta*; Co, *Celastrus orbiculatus*; Hh, *Hydrangea hydrangeoides*; Hp, *Hydrangea petiolaris*; Ta, *Trachelospermum asiaticum*; To, *Toxicodendron orientale*; Vc, *Vitis coignetiae*; Wf, *Wisteria floribunda*.

There were significant differences among climbing mechanisms and among species in all vessel traits (Fig. 5; Supplementary Data Fig. S6; Table S1). Because the tendril climbers in this study were composed of only one species, *V. coignetiae*, here we focused mainly on twining climbers and root climbers in the comparison of climbing mechanisms. The VD_mean of twining climbers was ∼1.7×, *D*_h_ was ∼2.3× and *K*_p_ was ∼7.3× those of the root climbers (Fig. 5; Supplementary Data Table S1). Root climbers had ∼4.2× the V_density of twining climbers, although the V_density values did not differ significantly among *C. orbiculatus*, *T. orientale* and *T. asiaticum*, despite the difference in climbing mechanisms. The V_fraction varied greatly within species and showed an overlap in its range among species. The *K*_p_ did not differ significantly within each climbing mechanism, although it differed clearly among climbing mechanisms. The VD_geomean and VD_max are shown in the Supplementary Data, because their patterns were similar to those of VD_mean and *D*_h_, respectively (Supplementary Data Fig. S7).

The vessels of species showing vessel dimorphism were classified into two clusters. The large-vessel cluster had a large variation in diameter and a small variation in Feret rate, whereas the small-vessel cluster had a small variation in diameter and a large variation in Feret rate (Fig. 4). The traits of the large-vessel clusters showed interspecific trends similar to those of the overall vessels (Supplementary Data Fig. S6). In addition, the *K*_p_ values of the large vessels were similar to those of the overall vessels. Therefore, the large vessels contributed most to the *K*_p_.

### Relationships between temperature and vessel traits

No consistent trends were found in the relationship between MAT and vessel traits within climbing mechanisms, although the relationships were detected in five of the eight species studied (Tables 3 and 4). A positive relationship between MAT and *K*_p_ was detected in *C. orbiculatus* and *H. hydrangeoides*. In *C. orbiculatus*, MAT was negatively associated with VD_mean and positively associated with VD_max, V_density and V_fraction (Table 3). Also, in *C. orbiculatus*, MAT had a positive relationship with *D*_h_, V_fraction and *K*_p_ in the large-vessel cluster and with V_density, V_fraction and *K*_p_ in the small vessel cluster (Table 4). Similar trends for small vessels were detected in *T. orientale*. In *H. hydrangeoides*, MAT was positively related to V_fraction, and in *A. arguta* this relationship was observed in the overall vessels and in the large-vessel cluster. In *T. asiaticum*, although the relationship between MAT and *K*_p_ was marginal (*P* = 0.056), MAT was associated positively with VD_mean, VD_max and *D*_h_ and negatively with V_density. Similar relationships were detected in the large-vessel cluster, whereas negative relationships were detected in the small-vessel cluster for V_density, V_fraction and *K*_p_ (Table 4). Stem diameter was associated positively with V_density in *C. orbiculatus* but negatively in *W. floribunda*. Stem diameter was also associated positively with *D*_h_ and *K*_p_ in *W. floribunda* (Table 3).

**Table 3.** Results of linear mixed-effects models examining the relationships between mean annual temperature and scaling of stem diameter, and vessel traits for all vessels.

Species	*N*	Exp	VD_mean	VD_max	*D*_h_	V_density	V_fraction	K_p_
**Aa**	157	SD	0.0148	0.1774*	0.1318	−0.2919	−0.2841	0.2210
		MAT	0.0003	−0.0013	0	0.0073	0.0177**	0.0070
**Co**	96	SD	−0.3297	0.0793	−0.0790	0.6117**	0.3316	0.2969
		MAT	−0.0209***	0.0094***	−0.0026	0.0389***	0.03**	0.0287***
**Wf**	64	SD	0.5120	0.2206	0.6344***	−0.8234**	0.8490	1.5629**
		MAT	−0.0042	−0.0010	−0.0048	−0.0013	−0.0220	−0.0179
**Hh**	158	SD	−0.0829	−0.1266	−0.0951	0.2041	0.0533	−0.1555
		MAT	0.0049	0.0032	0.0042	−0.0010	0.0199***	0.0161**
**Hp**	117	SD	0.0318	0.0161	0.0175	−0.1414	−0.2028	−0.0677
		MAT	0.0024	0.0004	0.0015	−0.0085	−0.0095	−0.0026
**To**	67	SD	0.2224	0.1269	0.2478	−0.1946	0.8282	0.6857
		MAT	−0.0077	0.0038	−0.002	0.0023	−0.0123	−0.0061
**Ta**	76	SD	0.0122	0.1295	0.0673	0.1508	0.4187	0.4201
		MAT	0.0186**	0.0139**	0.0173**	−0.0343***	0.0052	0.0348
**Vc**	101	SD	0.1555	0.0974	0.1437	−0.3545	0.0302	0.2101
		MAT	−0.0007	0.0005	−0.0004	0.0060	0.0100	0.0050

Columns of trait names indicate the partial regression coefficient estimated by the application of linear mixed-effects models to each variable. Significant values are indicated in bold. *P < 0.05; **P < 0.01; ***P < 0.001. Abbreviations: Aa, *Actinidia arguta*; Co, *Celastrus orbiculatus*; *D*_h_, mean hydraulically weighted vessel diameter; Hh, *Hydrangea hydrangeoides*; Hp, *Hydrangea petiolaris*; *K*_p_, potential hydraulic conductivity; MAT, mean annual temperature; *N*, sample size; SD, stem diameter; Ta, *Trachelospermum asiaticum*; To, *Toxicodendron orientale*; Vc, *Vitis coignetiae*; VD_max, maximum vessel diameter; VD_mean, mean vessel diameter; V_density, vessel density; V_fraction, vessel fraction; Wf, *Wisteria floribunda*.

**Table 4.** Results of linear mixed-effects models of relationships between of mean annual temperature and scaling of stem diameter and traits of each vessel cluster in species showing vessel dimorphism.

			Estimate									
			Small vessel cluster					Large vessel cluster				
Species	*N*	Exp	VD_mean	*D*_h_	V_density	V_fraction	*K*_p_	VD_mean	*D*_h_	V_density	V_fraction	*K*_p_
**Aa**	**157**	SD	0.0398	0.0383	−0.1608	−0.2109	−0.0190	0.1259	0.1757	−0.4124*	−0.2675	0.2248
		MAT	−0.0012	−0.0012	0.0065	0.0076	0.0013	0.0001	−0.0001	0.0086*	0.0179**	0.0071
**Co**	**96**	SD	0.0507	0.0696*	0.9943*	2.1556**	1.2690**	−0.0525	0.0308	0.2002	0.2609	0.2965
		MAT	0.0003	0	0.0645***	0.1361***	0.0646***	0.0046	0.0075**	−0.0021	0.0241*	0.0287***
**Wf**	**64**	SD	−0.4177***	−0.5524***	0.8073	−0.1466	−1.4081	0.8552**	0.7008***	−1.0764**	0.8638	1.5637**
		MAT	0.0002	0.0012	0.0014	0.0032	0.0062	−0.0069	−0.0059	−0.0001	−0.0222	−0.0179
**To**	**67**	SD	0.0126	0.0091	−0.4331	−0.7456	−0.3447	0.2049	0.1977	0.0262	1.0584	0.7093
		MAT	0.0003	0.0002	0.0236*	0.0458*	0.0243	0.0011	0.0026	−0.0178	−0.0240	−0.0076
**Ta**	**76**	SD	−0.0283	−0.0178	0.5646	0.9848	0.4934	0.0863	0.0987	0.0234	0.3880	0.4181
		MAT	−0.0023	−0.0030	−0.0788***	−0.1545***	−0.0908***	0.0142**	0.0145**	−0.0217**	0.0139	0.0363
**Vc**	**101**	SD	−0.0137	−0.0046	−0.444	−0.8805	−0.4306	0.0555	0.0701	−0.0459	0.1707	0.2134
		MAT	−0.0016	−0.0021	0.0068	0.0064	−0.0035	0.0017	0.0010	0.0019	0.0095	0.0051

Columns of each trait name indicate the partial regression coefficient obtained from application of the linear mixed-effects models to each variable. Significant values are indicated in bold. *P < 0.05; **P < 0.01; ***P < 0.001. Abbreviations: Aa, *Actinidia arguta*; Co, *Celastrus orbiculatus*; *D*_h_, mean hydraulically weighted vessel diameter; Exp, explanatory variable; *K*_p_, potential hydraulic conductivity; MAT, mean annual temperature; *N*, sample size; SD, stem diameter; Ta, *Trachelospermum asiaticum*; To, *Toxicodendron orientale*; Vc, *Vitis coignetiae*; V_density, vessel density; V_fraction, vessel fraction; VD_mean, mean vessel diameter; Wf, *Wisteria floribunda*.

## DISCUSSION

By comparing vessel traits and analysing the relationships between the traits and MAT in eight temperate liana species in Japan, this study indicated divergence in vessel traits among climbing mechanisms. In addition, variations in vessel traits in relationship to MAT were observed in some species.

### Differences in vessel traits between climbing mechanisms

Twining climbers and root climbers differed in all traits of overall vessels except for V_fraction. In addition, *K*_p_ varied by climbing mechanism in the overall vessels and in the large-vessel cluster. This difference in *K*_p_ among climbing mechanisms in this study was probably attributable to a difference in large-vessel diameter, because VD_max and *D*_h_ for overall vessels and VD_mean and *D*_h_ for large vessels showed trends similar to those of *K*_p_, namely, they were greater in twining climbers than in root climbers. The higher V_density in root climbers than in twining climbers seemed insufficient to match the contribution of vessel size in the twining climbers to *K*_p_. These results support hypothesis 1, that twining climbers have larger vessel diameters and greater water transport capacity than root climbers. The results also suggest that, because of their larger diameter, the vessels of twining climbers and *V. coignetiae* are at greater risk of freeze–thaw embolism (Davis *et al*., 1999; Tyree and Zimmermann, 2002) than those of root climbers with a similar stem diameter. However, not all vessels are functional in natural conditions. The proportion of non-functional vessels varies within and among species owing to the difference in the growth conditions and strategy of water transport (Utsumi *et al*., 1998, 1999; Davis *et al*., 1999; Niu *et al*., 2017), although *K*_p_ in this study assumed that all measured vessels were functional.

We propose several potential mechanisms to explain the differences in large-vessel diameter among the climbing mechanisms. The first is the difference in the relationship between stem diameter and stem length between climbing mechanisms (Ichihashi and Tateno, 2015). Root climbers might have a shorter length from the stem tip for a given stem diameter than twining climbers and *V. coignetiae*. Twining climbers and tendril climbers often hang from host trees, and after reaching the canopy they expand laterally to acquire additional hosts (Ichihashi and Tateno, 2011). In contrast, root climbers climb along a single host trunk and generally do not transfer to other hosts (Darwin, 1865; Ichihashi and Tateno, 2011). These ecological strategies might be reflected in the relationship between stem diameter and stem length of each climbing mechanism. Consequently, in this study, which compared vessel traits in samples of similar stem diameter, the difference in vessel size might have been observed via the influence of the path length. The second mechanism is the difference in water flow demand per unit stem diameter. If the amount of leaves per stem diameter is smaller, the rate of water flow per unit time required for that stem diameter would be reduced. In addition, different climbing mechanisms are likely to experience varying levels of transpiration demand owing to differences in their foliage display environment (Castellanos *et al*., 1992; Ishii *et al*., 2004; Avalos *et al*., 2007; Ichihashi and Tateno, 2011). Root climbers tend to display their foliage primarily in shaded environments, where the transpiration demand would be lower; they might therefore use a more conservative water transport strategy than twining climbers and *V. coignetiae*, which tend to display their foliage in well-lit environments (Ishii *et al*., 2004; Ichihashi and Tateno, 2011).

### Variations in vessel traits in relationship to MAT, and the interspecific differences

The *K*_p_ and vessel diameter decreased with decreasing MAT in some species, partly supporting hypothesis 2, that the diameter and density of large vessels and the fraction of all vessels decrease with decreasing temperature, and therefore the water transport capacity also decreases. However, because *K*_p_ varied inconsistently among species within the same climbing mechanisms, hypothesis 3, that twining climbers have greater changes in vessel diameter and water transport capacity along a temperature gradient than root climbers, was not supported.

The decrease in *K*_p_ with decreasing MAT in some species was attributed to differences in the changes of xylem structures in each species. In *C. orbiculatus*, changes in the size of the large vessels contributed to the change in *K*_p_, as indicated by the relationship of MAT to the VD_mean and *D*_h_ of large vessels and to VD_max, although the relationship with VD_mean of large vessels was marginal (*P* = 0.084). In addition, in *H. hydrangeoides* and *T. asiaticum* the reduction in the diameter of overall vessels with MAT led to a reduction in *K*_p_, although the relationships between MAT and VD_mean in *H. hydrangeoides* and *K*_p_ in *T. asiaticum* were marginal (*P* = 0.083 for VD_mean in *H. hydrangeoides* and *P* = 0.056 for *K*_p_ in *T. asiaticum*).

There might be several reasons for the relationship between MAT and vessel diameter in some species. The first is the direct effect of MAT on vessel diameter. Several studies have reported positive relationships between MAT and conduit diameter and hydraulically weighted conduit diameter (Fisher *et al*., 2007; Medeiros and Pockman, 2014; Hacke *et al*., 2017; Lobos-Catalán and Jiménez-Castillo, 2023), even when path length is considered (Olson *et al*., 2018). Natural selection by changes in transpiration demand with changing vapour pressure deficit (Olson *et al*., 2020) and by freeze–thaw embolism (Davis *et al*., 1999; Lobos-Catalán and Jiménez-Castillo, 2023) are assumed to be the factors underlying these relationships. The second reason is the indirect effect of MAT on vessel diameter via the allometric relationship between path length and stem diameter (Trouvé *et al*., 2015; Qiu *et al*., 2021). Here, we used stem diameter as a proxy for path length. Therefore, vessel diameter should change with MAT when the allometric relationship between path length and stem diameter is related to MAT. Particularly in lianas, reduced stem length for a given stem diameter might lead to a reduction in their growth and survival by requiring more resource allocation to gain stem length. This is because lianas are considered to invest excess resources in stem elongation and other organs by reducing their investment in supporting tissues (Wyka *et al*., 2013). Either of these factors could affect individual competitiveness.

Changes in the V_fraction would also contribute to variations in *K*_p_. The V_fraction decreased with decreasing MAT in *A. arguta*, *C. orbiculatus* and *H. hydrangeoides*. This trend suggests that there was an increase in abundance of the non-conductive tissues of the xylem in response to environmental stresses, such as an increase in parenchyma and storage of non-structural carbohydrates to cope with low temperatures (Chen *et al*., 2020; Yin *et al*., 2022). Xylem provides not only water transport pathways but also other functions, such as mechanical support and stress tolerance and response, and therefore contains associated tissues (Pratt and Jacobsen, 2017). Some species with the ability to refill embolized vessels contain large amounts of non-structural carbohydrates, mainly in the parenchyma, and these non-structural carbohydrates are linked to recovery from embolism (Chen *et al*., 2020; Yin *et al*., 2022).

In *A. arguta*, *C. orbiculatus* and *T. orientale*, vessels in different clusters responded variably to MAT. In *A. arguta*, a reduction in V_density with declining MAT was observed only in the large-vessel cluster, and this change was probably linked to a reduction in the V_fraction with declining MAT. In *C. orbiculatus*, a negative relationship between MAT and VD_mean was observed in the vessels overall. This trend differed in sign from previous findings within species (Medeiros and Pockman, 2014; Schreiber *et al*., 2015) and in cross-species analyses on a global scale (Olson *et al*., 2018). However, in *C. orbiculatus*, the trend would have occurred because the increase in the density and fraction of small vessels outweighed that of large vessels. Likewise, in *T. orientale*, the density and fraction of small vessels increased with increasing MAT. To our knowledge, no previous studies have reported changes in the density of small vessels, although, consistent with our results, it has been observed that the diameters of small vessels in some species remain stable in relationship to environmental changes (Schreiber *et al*., 2015; García-Cervigón *et al*., 2020). The decrease in the density of small vessels with decreasing temperature was contrary to our expectation, given the function of small vessels in ensuring safe water transport (Carlquist, 2012; Jiménez-Castillo and Lusk, 2013). Therefore, this pattern and its mechanisms should be confirmed. Note that changes in small vessel density might result from errors in vessel detection during image processing. If the size of cells other than the vessels increase in warm conditions, or if the number of vessels smaller than the threshold increases in cold environments, it might lead to the false detection of cell hollows and vessels. However, even if this error affects the trends in small vessel abundance, the relationship between *K*_p_ and MAT remains robust in terms of our results for the large-vessel traits.

Although relationships of MAT to vessel traits were detected in five of the eight species studied, approximately half of the species showed no relationship between MAT and vessel traits. This absence of patterns could result from compensatory mechanisms occurring in systems other than the xylem, such as the ability to refill embolized vessels by using positive pressure generated by the roots (Tibbetts and Ewers, 2000; Améglio *et al*., 2002; Schenk *et al*., 2021). In addition, the relationships between stem diameter and the traits of all vessels were generally non-significant, except in the cases of V_density in *C. orbiculatus*, *D*_h_, V_density and V_fraction in *W. floribunda*, and VD_max in *A. arguta*. Moreover, the estimated partial regression coefficients for these species deviated from those reported previously (Rosell and Olson, 2014). These discrepancies were probably attributable to the limited range of stem diameters examined here or the relatively large errors in the stem diameter–path length relationship in lianas (Rosell and Olson, 2014), or both.

### Limitations of the methodology used for vessel classification

We classified vessels by GMMs using the vessel diameter and Feret rate as variables. The analyses revealed changes in the size distribution of vessels with changes in MAT in some species by indicating different relationships between each cluster and MAT. However, this classification does not always accurately reflect functional differences. In fact, some vessels that showed small diameter seemed to be classified into the large-vessel clusters in some species (e.g. *C. orbiculatus*, *T. orientale* and *V. coignetiae*; Fig. 4). As a result, the mean vessel diameter and the vessel density of large vessels would have been underestimated and overestimated, respectively. Furthermore, we set a threshold of 30 μm for vessel diameter to avoid misidentifying non-vessel hollows as vessels. As a result, vessels with diameters below the threshold were not evaluated, and evaluation of the response of small vessels might be obscure. Indeed, several studies that examined xylem structure in lianas with similar stem diameters have reported the presence of vessels <30 μm in diameter (Gerolamo and Angyalossy, 2017; Chery et al., 2020; Gerolamo *et al*., 2020). Despite this inaccuracy, in large vessels of these species, the VD_mean was larger and the V_density smaller than that in root climbers. Furthermore, the relationships between vessel traits and MAT were still supported, because the same classification criteria were applied regardless of sampling site for each species. This vessel classification allowed statistical testing of the relationships between environmental factors and vessels of different sizes, although more research is needed to develop more appropriate methods of vessel classification to consider and understand the functional differences among vessels.

### Conclusion

We showed here that the vessel traits in temperate lianas differed by climbing mechanism. In addition, we revealed a decrease in potential water transport capacity with decreasing MAT, and structural changes in the xylem that contributed to water transport capacity in some species. Furthermore, vessels of different sizes exhibited distinct relationships to MAT. The observed differences in vessel traits between the two climbing mechanisms were consistent with the distribution patterns of the two climbing mechanisms along the temperature gradient, as found by Kusakabe *et al*. (2023). Therefore, our results would partly explain the distribution patterns of these two climbing mechanisms in this region. The observed reduction in *K*_p_ with decreasing MAT in some species suggests a reduction in the performance of individual lianas, although it remains unclear whether this reduction in *K*_p_ is directly linked to freeze–thaw embolism. This trend supports the prediction that liana competitiveness decreases with decreasing temperature (Schnitzer, 2005). Nevertheless, the relationships of vessel traits to MAT varied by species regardless of the climbing mechanism. These interspecific variations might be related to differences in the performance of species in a changing environment. Therefore, understanding these variations in plant hydraulic function in future studies will provide fundamental information for predicting changes in forest community structure and ecosystem functions, such as transpiration and carbon storage, in response to environmental change.

## Supplementary Material

mcaf138_Supplementary_Data

## Supplementary Data

Supplementary data are available at *Annals of Botany*  online and consistent of the following. Figure S1: sampling sites of each liana species. Figure S2: determination of the Feret rate. Figure S3: relationships between mean annual temperature and freeze–thaw indices. Figure S4: relationships between the stem diameter of sampled individuals and mean annual temperature at the sampling locations. Figure S5: differences in stem diameter among climbing mechanisms and among liana species. Figure S6: differences in xylem vessel traits among climbing mechanisms and among species in each vessel cluster of lianas. Figure S7: supplementary results for vessel trait comparisons among climbing mechanisms and among species of lianas. Table S1: representative values of traits in liana species in the study.

## References

[mcaf138-B1] Améglio T, Bodet C, Lacointe A, Cochard H. 2002. Winter embolism, mechanisms of xylem hydraulic conductivity recovery and springtime growth patterns in walnut and peach trees. Tree Physiology 22: 1211–1220. doi:10.1093/treephys/22.17.1211 12464574

[mcaf138-B2] Anderegg WRL, Konings AG, Trugman AT, et al 2018. Hydraulic diversity of forests regulates ecosystem resilience during drought. Nature 561: 538–541. doi:10.1038/s41586-018-0539-7 30232452

[mcaf138-B3] Avalos G, Mulkey SS, Kitajima K, Wright SJ. 2007. Colonization strategies of two liana species in a tropical dry forest canopy. Biotropica 39: 393–399. doi:10.1111/j.1744-7429.2007.00265.x

[mcaf138-B4] Barik SK, Adhikari D, Chettri A, Singh PP. 2015. Diversity of lianas in eastern Himalayas and north-eastern India. In: Parthasarathy N. ed. Biodiversity of lianas. Cham, Switzerland: Springer, 99–121.

[mcaf138-B5] Brooks ME, Kristensen K, van Benthem KJ, et al 2017. glmmTMB balances speed and flexibility among packages for zero-inflated generalized linear mixed modeling. The R Journal 9: 378–400. doi:10.32614/RJ-2017-066

[mcaf138-B6] Carlquist S . 1981. Wood anatomy of Nepenthaceae. Bulletin of the Torrey Botanical Club 108: 324–330. doi:10.2307/2484711

[mcaf138-B7] Carlquist S . 1985. Observations on functional wood histology of vines and lianas. A Journal of Systematic and Floristic Botany 11: 139–157. doi:10.5642/aliso.19851102.03

[mcaf138-B8] Carlquist S . 2012. How wood evolves: a new synthesis. Botany 90: 901–940. doi:10.1139/b2012-048

[mcaf138-B9] Castagneri D, Garbarino M, Nola P. 2013. Host preference and growth patterns of ivy (*Hedera helix* L.) in a temperate alluvial forest. Plant Ecology 214: 1–9. doi:10.1007/s11258-012-0130-5

[mcaf138-B10] Castellanos AE, Duran R, Guzman S, Briones O, Feria M. 1992. Three-dimensional space utilization of lianas: a methodology. Biotropica 24: 396. doi:10.2307/2388609

[mcaf138-B11] Chen Z, Zhu S, Zhang Y, et al 2020. Tradeoff between storage capacity and embolism resistance in the xylem of temperate broadleaf tree species. Tree Physiology 40: 1029–1042. doi:10.1093/treephys/tpaa046 32310276

[mcaf138-B12] Chery JG, Neto IdC, Pace MR, Acevedo-Rodríguez P, Specht CD, Rothfels CJ. 2020. Wood anatomy of the neotropical liana lineage *Paullinia* L. (Sapindaceae). IAWA Journal 41: 278–300. doi:10.1163/22941932-bja10027

[mcaf138-B13] Darwin C . 1865. On the movements and habits of climbing plants. The Journal of the Linnean Society. Botany 9: 1–118. doi:10.1111/j.1095-8339.1865.tb00011.x

[mcaf138-B14] Davis SD, Sperry JS, Hacke UG. 1999. The relationship between xylem conduit diameter and cavitation caused by freezing. American Journal of Botany 86: 1367–1372. doi:10.2307/2656919 10523278

[mcaf138-B15] Dias AS, Oliveira RS, Martins FR, Bongers F, Anten NPR, Sterck FJ. 2024. Climbing mechanisms as a central trait to understand the ecology of lianas across the tropics. Global Ecology and Biogeography 33: e13846. doi:10.1111/geb.13846

[mcaf138-B16] Dormann CF, Elith J, Bacher S, et al 2013. Collinearity: a review of methods to deal with it and a simulation study evaluating their performance. Ecography 36: 27–46. doi:10.1111/j.1600-0587.2012.07348.x

[mcaf138-B17] Fajardo A, Piper FI, García-Cervigón AI. 2022. The intraspecific relationship between wood density, vessel diameter and other traits across environmental gradients. Functional Ecology 36: 1585–1598. doi:10.1111/1365-2435.14069

[mcaf138-B18] Fisher JB, Goldstein G, Jones TJ, Cordell S. 2007. Wood vessel diameter is related to elevation and genotype in the Hawaiian tree *Metrosideros polymorpha* (Myrtaceae). American Journal of Botany 94: 709–715. doi:10.3732/ajb.94.5.709 21636440

[mcaf138-B19] Flo V, Martínez-Vilalta J, Mencuccini M, Granda V, Anderegg WRL, Poyatos R. 2021. Climate and functional traits jointly mediate tree water-use strategies. The New Phytologist 231: 617–630. doi:10.1111/nph.17404 33893652

[mcaf138-B20] García-Cervigón AI, Fajardo A, Caetano-Sánchez C, Camarero JJ, Olano JM. 2020. Xylem anatomy needs to change, so that conductivity can stay the same: xylem adjustments across elevation and latitude in *Nothofagus pumilio*. Annals of Botany 125: 1101–1112. doi:10.1093/aob/mcaa042 32173741 PMC7262467

[mcaf138-B21] García-Cervigón AI, Olano JM, von Arx G, Fajardo A. 2018. Xylem adjusts to maintain efficiency across a steep precipitation gradient in two coexisting generalist species. Annals of Botany 122: 461–472. doi:10.1093/aob/mcy088 29800073 PMC6110345

[mcaf138-B22] Gerolamo CS, Angyalossy V. 2017. Wood anatomy and conductivity in lianas, shrubs and trees of Bignoniaceae. IAWA Journal 38: 412–432. doi:10.1163/22941932-20170177

[mcaf138-B23] Gerolamo CS, Nogueira A, Pace MR, Angyalossy V. 2020. Interspecific anatomical differences result in similar highly flexible stems in Bignoniaceae lianas. American Journal of Botany 107: 1622–1634. doi:10.1002/ajb2.1577 33274437

[mcaf138-B24] Gianoli E . 2015. Evolutionary implications of the climbing habit in plants. In: Schnitzer SA, Bongers F, Burnham RJ, Putz FE. eds. Ecology of lianas. Chichester, UK: John Wiley & Sons, 239–250.

[mcaf138-B25] Gianoli E, Saldaña A, Jiménez-Castillo M, Valladares F. 2010. Distribution and abundance of vines along the light gradient in a southern temperate rain forest. Journal of Vegetation Science 21: 66–73. doi:10.1111/j.1654-1103.2009.01124.x

[mcaf138-B26] Gleason SM, Westoby M, Jansen S, et al 2016. Weak tradeoff between xylem safety and xylem-specific hydraulic efficiency across the world’s woody plant species. The New Phytologist 209: 123–136. doi:10.1111/nph.13646 26378984

[mcaf138-B27] Hacke UG, Spicer R, Schreiber SG, Plavcová L. 2017. An ecophysiological and developmental perspective on variation in vessel diameter. Plant, Cell and Environment 40: 831–845. doi:10.1111/pce.12777 27304704

[mcaf138-B28] Hothorn T, Bretz F, Westfall P. 2008. Simultaneous inference in general parametric models. Biometrical Journal 50: 346–363. doi:10.1002/bimj.200810425 18481363

[mcaf138-B29] Hu L, Li M, Li Z. 2010. Geographical and environmental gradients of lianas and vines in China. Global Ecology and Biogeography 19: 554–561. doi:10.1111/j.1466-8238.2010.00527.x

[mcaf138-B30] Ichihashi R, Chiu C, Komatsu H, et al 2017. Contribution of lianas to community-level canopy transpiration in a warm-temperate forest. Functional Ecology 31: 1690–1699. doi:10.1111/1365-2435.12881

[mcaf138-B31] Ichihashi R, Tateno M. 2011. Strategies to balance between light acquisition and the risk of falls of four temperate liana species: to overtop host canopies or not? The Journal of Ecology 99: 1071–1080. doi:10.1111/j.1365-2745.2011.01808.x

[mcaf138-B32] Ichihashi R, Tateno M. 2015. Biomass allocation and long-term growth patterns of temperate lianas in comparison with trees. The New Phytologist 207: 604–612. doi:10.1111/nph.13391 25817272

[mcaf138-B33] Isasa E, Link RM, Jansen S, et al 2023. Addressing controversies in the xylem embolism resistance–vessel diameter relationship. The New Phytologist 238: 283–296. doi:10.1111/nph.18731 36636783

[mcaf138-B34] Ishihara MI, Suzuki SN, Nakamura M, et al 2011. Forest stand structure, composition, and dynamics in 34 sites over Japan. Ecological Research 26: 1007–1008. doi:10.1007/s11284-011-0847-y

[mcaf138-B35] Ishii HT, Tanabe SI, Hiura T. 2004. Exploring the relationships among canopy structure, stand productivity, and biodiversity of temperate forest ecosystems. Forest Science 50: 342–355. doi:10.1093/forestscience/50.3.342

[mcaf138-B36] Isnard S, Feild TS. 2015. The evolution of angiosperm lianescence: a perspective from xylem structure-function. In: Schnitzer SA, Bongers F, Burnham RJ, Putz FE. eds. Ecology of lianas. Chichester, UK: John Wiley & Sons, 221–238.

[mcaf138-B37] Isnard S, Silk WK. 2009. Moving with climbing plants from Charles Darwin’s time into the 21st century. American Journal of Botany 96: 1205–1221. doi:10.3732/ajb.0900045 21628270

[mcaf138-B38] Janssens S, Couvreur TLP, Mertens A, et al 2020. A large-scale species level dated angiosperm phylogeny for evolutionary and ecological analyses. Biodiversity Data Journal 8: e39677. doi:10.3897/BDJ.8.e39677 32015666 PMC6987248

[mcaf138-B39] Jiménez-Castillo M, Lusk CH. 2013. Vascular performance of woody plants in a temperate rain forest: lianas suffer higher levels of freeze–thaw embolism than associated trees. Functional Ecology 27: 403–412. doi:10.1111/1365-2435.12045

[mcaf138-B40] Jiménez-Castillo M, Wiser SK, Lusk CH. 2007. Elevational parallels of latitudinal variation in the proportion of lianas in woody floras. Journal of Biogeography 34: 163–168. doi:10.1111/j.1365-2699.2006.01570.x

[mcaf138-B41] Kusakabe G, Mori H, Hiura T. 2023. Distribution patterns of lianas from subtropical to subboreal zones of the Japanese archipelago and the difference between climbing types. Basic and Applied Ecology 72: 1–9. doi:10.1016/j.baae.2023.08.001

[mcaf138-B42] Lachenbruch B, McCulloh KA. 2014. Traits, properties, and performance: how woody plants combine hydraulic and mechanical functions in a cell, tissue, or whole plant. The New Phytologist 204: 747–764. doi:10.1111/nph.13035 25250668

[mcaf138-B43] Lobos-Catalán P, Jiménez-Castillo M. 2019. Temperature shapes liana diversity pattern along a latitudinal gradient in southern temperate rainforest. Plant Ecology 220: 1109–1117. doi:10.1007/s11258-019-00980-7

[mcaf138-B44] Lobos-Catalán P, Jiménez-Castillo M. 2023. The functional mechanism behind the latitudinal pattern of liana diversity: freeze–thaw embolism reduces the ecological performance of liana species. Ecology and Evolution 13: e10486. doi:10.1002/ece3.10486 37736281 PMC10509155

[mcaf138-B45] Londré RA, Schnitzer SA. 2006. The distribution of lianas and their change in abundance in temperate forests over the past 45 years. Ecology 87: 2973–2978. doi:10.1890/0012-9658(2006)87[2973:TDOLAT]2.0.CO;2 17249220

[mcaf138-B46] McDowell NG, Brodribb TJ, Nardini A. 2019. Hydraulics in the 21^st^ century. The New Phytologist 224: 537–542. doi:10.1111/nph.16151 31545889

[mcaf138-B47] Medeiros JS, Pockman WT. 2014. Freezing regime and trade-offs with water transport efficiency generate variation in xylem structure across diploid populations of *Larrea* sp. (Zygophyllaceae). American Journal of Botany 101: 598–607. doi:10.3732/ajb.1400046 24699537

[mcaf138-B48] Morris H, Plavcová L, Cvecko P, et al 2016. A global analysis of parenchyma tissue fractions in secondary xylem of seed plants. The New Phytologist 209: 1553–1565. doi:10.1111/nph.13737 26551018 PMC5063116

[mcaf138-B49] Nakaba S, Kitin P, Yamagishi Y, et al 2015. Three-dimensional imaging of cambium and secondary xylem cells by confocal laser scanning microscopy. In: Yeung ECT, Stasolla C, Summer MJ, Huang BQ. eds. Plant microtechniques: methods and protocols. Heidelberg, Germany: Springer, 431–465.

[mcaf138-B50] Niu C, Meinzer FC, Hao G. 2017. Divergence in strategies for coping with winter embolism among co-occurring temperate tree species: the role of positive xylem pressure, wood type and tree stature. Functional Ecology 31: 1550–1560. doi:10.1111/1365-2435.12868

[mcaf138-B51] Ohno H, Sasaki K, Ohara G, Nakazono K. 2016. Development of grid square air temperature and precipitation data compiled from observed, forecasted, and climatic normal data [in Japanese with English summary]. Climate in Biosphere 16: 71–79. doi:10.2480/cib.J-16-028

[mcaf138-B52] Olson ME . 2022. Linking xylem structure and function: the comparative method in from the cold. The New Phytologist 235: 815–820. doi:10.1111/nph.18179 35770485 PMC9328200

[mcaf138-B53] Olson ME, Anfodillo T, Gleason SM, McCulloh KA. 2021. Tip-to-base xylem conduit widening as an adaptation: causes, consequences, and empirical priorities. The New Phytologist 229: 1877–1893. doi:10.1111/nph.16961 32984967

[mcaf138-B54] Olson ME, Anfodillo T, Rosell JA, Martínez-Méndez N. 2020. Across climates and species, higher vapour pressure deficit is associated with wider vessels for plants of the same height. Plant, Cell and Environment 43: 3068–3080. doi:10.1111/pce.13884 32909290

[mcaf138-B55] Olson ME, Rosell JA. 2013. Vessel diameter–stem diameter scaling across woody angiosperms and the ecological causes of xylem vessel diameter variation. The New Phytologist 197: 1204–1213. doi:10.1111/nph.12097 23278439

[mcaf138-B56] Olson ME, Rosell JA, León C, et al 2013. Convergent vessel diameter–stem diameter scaling across five clades of New and Old World eudicots from desert to rain forest. International Journal of Plant Sciences 174: 1062–1078. doi:10.1086/671432

[mcaf138-B57] Olson ME, Soriano D, Rosell JA, et al 2018. Plant height and hydraulic vulnerability to drought and cold. Proceedings of the National Academy of Sciences of the United States of America 115: 7551–7556. doi:10.1073/pnas.1721728115 29967148 PMC6055177

[mcaf138-B58] Parthasarathy N, Vivek P, Muthumperumal C, Muthuramkumar S, Ayyappan N. 2015. Biodiversity of lianas and their functional traits in tropical forests of peninsular India. In: Parthasarathy N. ed. Biodiversity of Lianas. Cham, Switzerland: Springer, 123–148.

[mcaf138-B59] Pellissari LCO, Barros CF, Medeiros H, Tamaio N. 2018. Cambial patterns of *Paullinia* (Sapindaceae) in southwestern Amazonia, Brazil. Flora 246–247: 71–82. doi:10.1016/j.flora.2018.07.002

[mcaf138-B60] Péros J-P, Berger G, Portemont A, Boursiquot J-M, Lacombe T. 2011. Genetic variation and biogeography of the disjunct *Vitis* subg. *Vitis* (Vitaceae). Journal of Biogeography 38: 471–486. doi:10.1111/j.1365-2699.2010.02410.x

[mcaf138-B61] Poorter L, McDonald I, Alarcón A, et al 2010. The importance of wood traits and hydraulic conductance for the performance and life history strategies of 42 rainforest tree species. The New Phytologist 185: 481–492. doi:10.1111/j.1469-8137.2009.03092.x 19925555

[mcaf138-B62] Pratt RB, Jacobsen AL. 2017. Conflicting demands on angiosperm xylem: tradeoffs among storage, transport and biomechanics. Plant, Cell and Environment 40: 897–913. doi:10.1111/pce.12862 27861981

[mcaf138-B63] Qiu H, Liu S, Zhang Y, Li J. 2021. Variation in height-diameter allometry of ponderosa pine along competition, climate, and species diversity gradients in the western United States. Forest Ecology and Management 497: 119477. doi:10.1016/j.foreco.2021.119477

[mcaf138-B64] R Core Team . 2024. *R: a language and environment for statistical computing*. Vienna, Austria: R Foundation for Statistical Computing. https://www.R-project.org

[mcaf138-B65] Rosell JA, Olson ME. 2014. Do lianas really have wide vessels? Vessel diameter–stem length scaling in non-self-supporting plants. Perspectives in Plant Ecology, Evolution and Systematics 16: 288–295. doi:10.1016/j.ppees.2014.08.001

[mcaf138-B66] Rosell JA, Olson ME, Anfodillo T. 2017. Scaling of xylem vessel diameter with plant size: causes, predictions, and outstanding questions. Current Forestry Reports 3: 46–59. doi:10.1007/s40725-017-0049-0

[mcaf138-B67] Savage VM, Bentley LP, Enquist BJ, et al 2010. Hydraulic trade-offs and space filling enable better predictions of vascular structure and function in plants. Proceedings of the National Academy of Sciences of the United States of America 107: 22722–22727. doi:10.1073/pnas.1012194108 21149696 PMC3012458

[mcaf138-B68] Schenk HJ, Jansen S, Hölttä T. 2021. Positive pressure in xylem and its role in hydraulic function. The New Phytologist 230: 27–45. doi:10.1111/nph.17085 33206999

[mcaf138-B69] Schindelin J, Arganda-Carreras I, Frise E, et al 2012. Fiji: an open-source platform for biological-image analysis. Nature Methods 9: 676–682. doi:10.1038/nmeth.2019 22743772 PMC3855844

[mcaf138-B70] Schnitzer SA . 2005. A mechanistic explanation for global patterns of liana abundance and distribution. The American Naturalist 166: 262–276. doi:10.1086/431250 16032578

[mcaf138-B71] Schnitzer SA . 2018. Testing ecological theory with lianas. The New Phytologist 220: 366–380. doi:10.1111/nph.15431 30247750

[mcaf138-B72] Schreiber SG, Hacke UG, Hamann A. 2015. Variation of xylem vessel diameters across a climate gradient: insight from a reciprocal transplant experiment with a widespread boreal tree. Functional Ecology 29: 1392–1401. doi:10.1111/1365-2435.12455

[mcaf138-B73] Scrucca L, Fop M, Murphy TB, Raftery AE. 2016. mclust 5: clustering, classification and density estimation using Gaussian finite mixture models. The R Journal 8: 289–317. 27818791 PMC5096736

[mcaf138-B74] Sperotto P, Acevedo-Rodríguez P, Vasconcelos TNC, Roque N. 2020. Towards a standardization of terminology of the climbing habit in plants. The Botanical Review 86: 180–210. doi:10.1007/s12229-020-09218-y

[mcaf138-B75] Sperotto P, Roque N, Acevedo-Rodríguez P, Vasconcelos T. 2023. Climbing mechanisms and the diversification of neotropical climbing plants across time and space. The New Phytologist 240: 1561–1573. doi:10.1111/nph.19093 37381080

[mcaf138-B76] Sperry JS, Meinzer FC, McCulloh KA. 2008. Safety and efficiency conflicts in hydraulic architecture: scaling from tissues to trees. Plant, Cell and Environment 31: 632–645. doi:10.1111/j.1365-3040.2007.01765.x 18088335

[mcaf138-B77] Steppe K, Lemeur R. 2007. Effects of ring-porous and diffuse-porous stem wood anatomy on the hydraulic parameters used in a water flow and storage model. Tree Physiology 27: 43–52. doi:10.1093/treephys/27.1.43 17169905

[mcaf138-B78] Sterck FJ, Zweifel R, Sass-Klaassen U, Chowdhury Q. 2008. Persisting soil drought reduces leaf specific conductivity in Scots pine (*Pinus sylvestris*) and pubescent oak (*Quercus pubescens*). Tree Physiology 28: 529–536. doi:10.1093/treephys/28.4.529 18244940

[mcaf138-B79] Thévenaz P, Unser M. 2007. User-friendly semiautomated assembly of accurate image mosaics in microscopy. Microscopy Research and Technique 70: 135–146. doi:10.1002/jemt.20393 17133410

[mcaf138-B80] Tibbetts TJ, Ewers FW. 2000. Root pressure and specific conductivity in temperate lianas: exotic *Celastrus orbiculatus* (Celastraceae) vs. native *Vitis riparia* (Vitaceae). American Journal of Botany 87: 1272–1278. doi:10.2307/2656720 10991898

[mcaf138-B81] Tröndle D, Schröder S, Kassemeyer H-H, Kiefer C, Koch MA, Nick P. 2010. Molecular phylogeny of the genus *Vitis* (Vitaceae) based on plastid markers. American Journal of Botany 97: 1168–1178. doi:10.3732/ajb.0900218 21616868

[mcaf138-B82] Trouvé R, Bontemps J-D, Seynave I, Collet C, Lebourgeois F. 2015. Stand density, tree social status and water stress influence allocation in height and diameter growth of *Quercus petraea* (Liebl.). Tree Physiology 35: 1035–1046. doi:10.1093/treephys/tpv067 26232785

[mcaf138-B83] Tyree MT, Zimmermann MH. 1971. The theory and practice of measuring transport coefficients and sap flow in the xylem of red maple stems (*Acer rubrum*). Journal of Experimental Botany 22: 1–18. doi:10.1093/jxb/22.1.1

[mcaf138-B84] Tyree MT, Zimmermann MH. 2002. Xylem structure and the ascent of sap. Berlin, Heidelberg, Germany: Springer.

[mcaf138-B85] Utsumi Y, Sano Y, Fujikawa S, Funada R, Ohtani J. 1998. Visualization of cavitated vessels in winter and refilled vessels in spring in diffuse-porous trees by cryo-scanning electron microscopy. Plant Physiology 117: 1463–1471. doi:10.1104/pp.117.4.1463 9701601 PMC34909

[mcaf138-B86] Utsumi Y, Sano Y, Funada R, Fujikawa S, Ohtani J. 1999. The progression of cavitation in earlywood vessels of *Fraxinus mandshurica* var *japonica* during freezing and thawing. Plant Physiology 121: 897–904. doi:10.1104/pp.121.3.897 10557238 PMC59452

[mcaf138-B87] van der Heijden GMF, Powers JS, Schnitzer SA. 2015. Lianas reduce carbon accumulation and storage in tropical forests. Proceedings of the National Academy of Sciences of the United States of America 112: 13267–13271. doi:10.1073/pnas.1504869112 26460031 PMC4629347

[mcaf138-B88] van der Heijden GM, Schnitzer SA, Powers JS, Phillips OL. 2013. Liana impacts on carbon cycling, storage and sequestration in tropical forests. Biotropica 45: 682–692. doi:10.1111/btp.12060

[mcaf138-B89] Venturas MD, Sperry JS, Hacke UG. 2017. Plant xylem hydraulics: what we understand, current research, and future challenges. Journal of Integrative Plant Biology 59: 356–389. doi:10.1111/jipb.12534 28296168

[mcaf138-B90] Warton DI, Hui FKC. 2011. The arcsine is asinine: the analysis of proportions in ecology. Ecology 92: 3–10. doi:10.1890/10-0340.1 21560670

[mcaf138-B91] West GB, Brown JH, Enquist BJ. 1999. A general model for the structure and allometry of plant vascular systems. Nature 400: 664–667. doi:10.1038/23251

[mcaf138-B92] Wickham H, Averick M, Bryan J, et al 2019. Welcome to the tidyverse. Journal of Open Source Software 4: 1686. doi:10.21105/joss.01686

[mcaf138-B93] Wyka TP, Oleksyn J, Karolewski P, Schnitzer SA. 2013. Phenotypic correlates of the lianescent growth form: a review. Annals of Botany 112: 1667–1681. doi:10.1093/aob/mct236 24169592 PMC3838560

[mcaf138-B94] Yin X, Hao G, Sterck F. 2022. A trade-off between growth and hydraulic resilience against freezing leads to divergent adaptations among temperate tree species. Functional Ecology 36: 739–750. doi:10.1111/1365-2435.13991

[mcaf138-B95] Zhang K-Y, Yang D, Zhang Y-B, et al 2023. Vessel dimorphism and wood traits in lianas and trees among three contrasting environments. American Journal of Botany 110: e16154. doi:10.1002/ajb2.16154 36912354

