# Supplementary material for: Are differences in xylem vessel traits and their geographical variation among liana species related to the distribution patterns of climbing mechanisms in a temperate zone?
Source: Ann Bot. 2025 Jul 10;137(1):79–94. doi: 10.1093/aob/mcaf138 (PMC12784078; doi:10.1093/aob/mcaf138)
Supplement: mcaf138_Supplementary_Data [file mcaf138_supplementary_data.docx]

**Supplementary information 1.**

**Supplementary figures.**

**
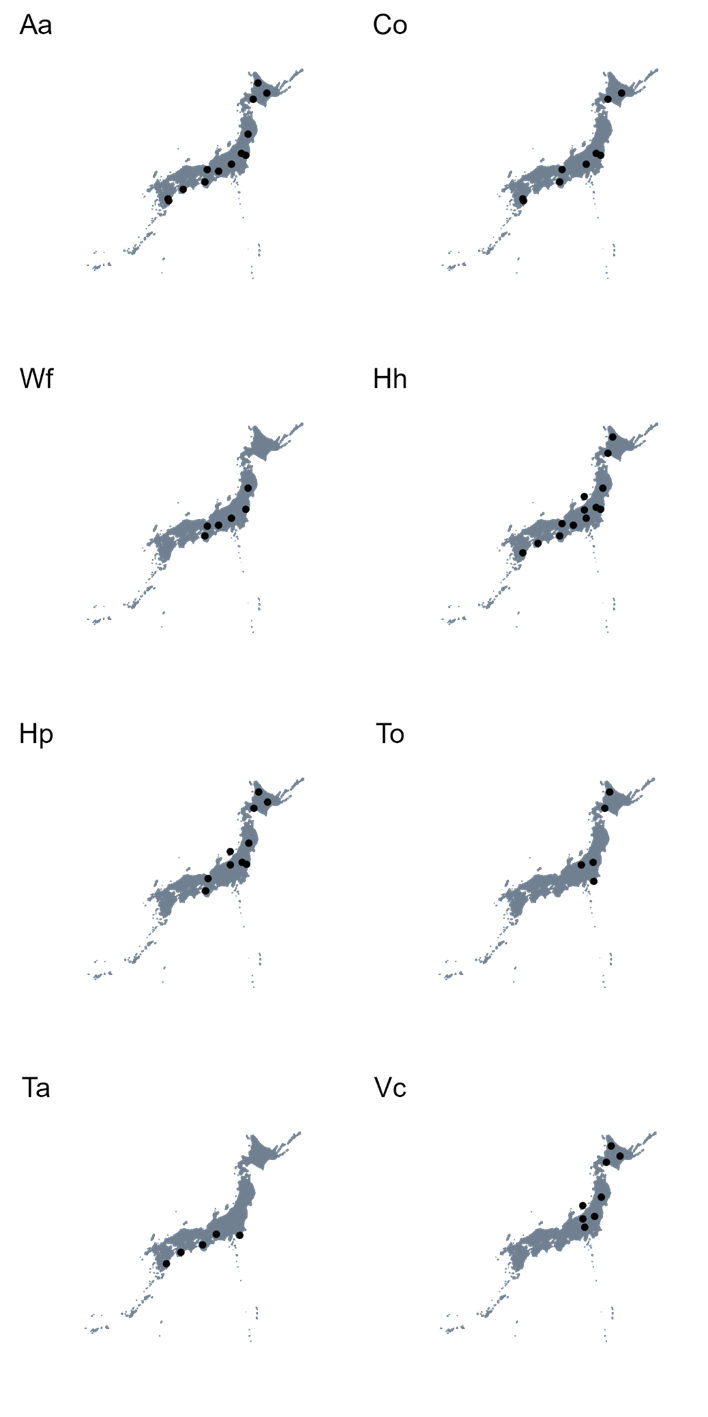
**

**Figure S1.** **Sampling sites of each liana species**

Filled circles indicate study sites. Aa, *Actinidia arguta*; Co, *Celastrus orbiculatus*; Wf, *Wisteria floribunda*; Hh, *Hydrangea hydrangeoides*; Hp, *Hydrangea petiolaris*; To, *Toxicodendron orientale*; Ta, *Trachelospermum asiaticum*; Vc, *Vitis coignetiae*.


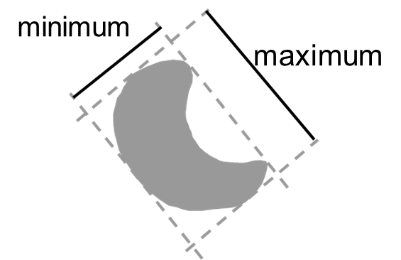


**Figure S2. Determination of the Feret rate**

Feret diameter is defined as the distance between the two parallel lines tangent to the outline of an object. In this study, the maximum and minimum values of feret diameter were used to calculate Feret rate of each vessel to capture the shapes of vessels.


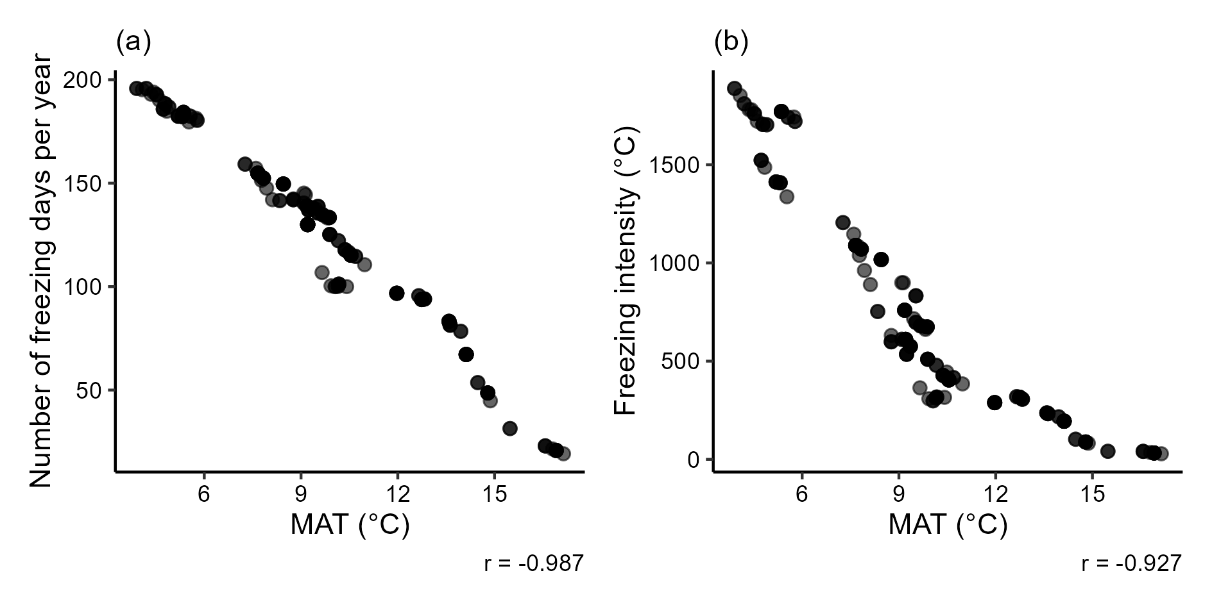


**Figure S3.** **Relationships between mean annual temperature and freeze–thaw indices**

Points indicate individuals studied. (a) Number of freezing days per year was calculated as the 5-year average of the annual total of days with a minimum daily temperature < 0 °C at the sample location. (b) Freezing intensity was calculated as the 5-year average of the sum of the absolute values of the daily minimum temperature on days with a minimum temperature < 0 °C. *r*, Pearson product-moment correlation coefficient. MAT, mean annual temperature.


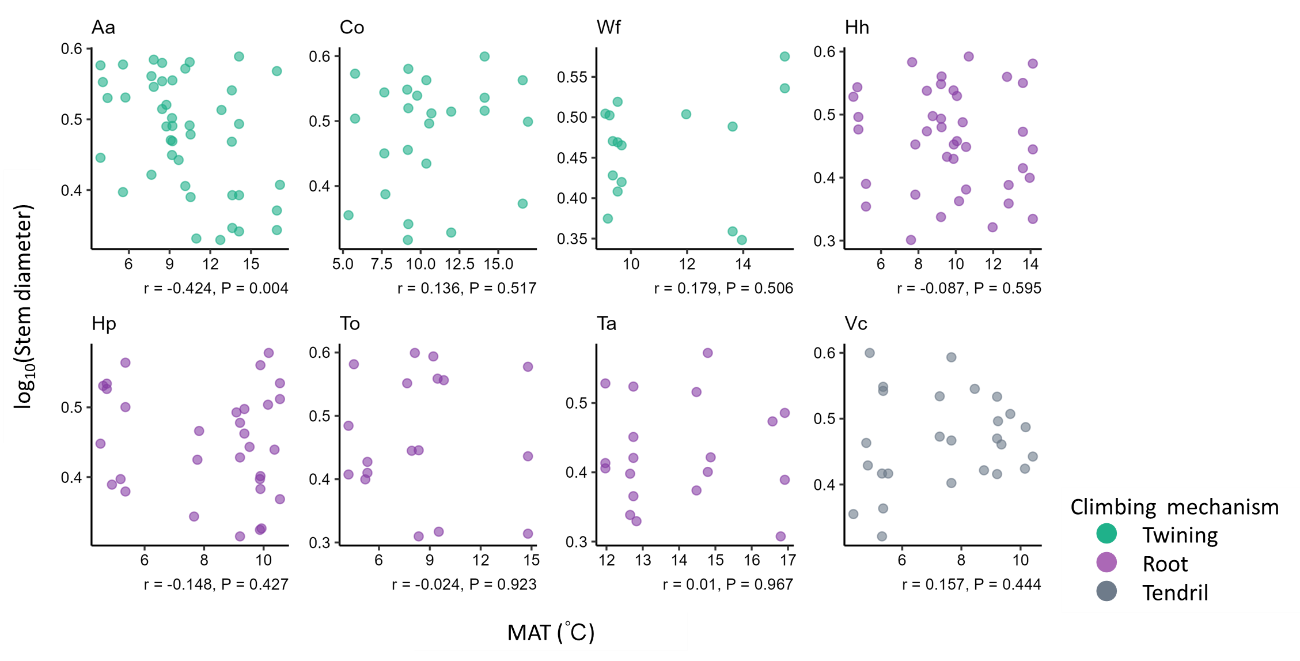


**Figure S4.** **Relationships between the stem diameter of sampled individuals and mean annual temperature at the sampling locations**

Points indicate individuals studied. *r*, Pearson product-moment correlation coefficient; p, *P*-value from a *t*-test based on the correlation coefficient. MAT, mean annual temperature; Aa, *Actinidia arguta*; Co, *Celastrus orbiculatus*; Wf, *Wisteria floribunda*; Hh, *Hydrangea hydrangeoides*; Hp, *Hydrangea petiolaris*; To, *Toxicodendron orientale*; Ta, *Trachelospermum asiaticum*; Vc, *Vitis coignetiae*.


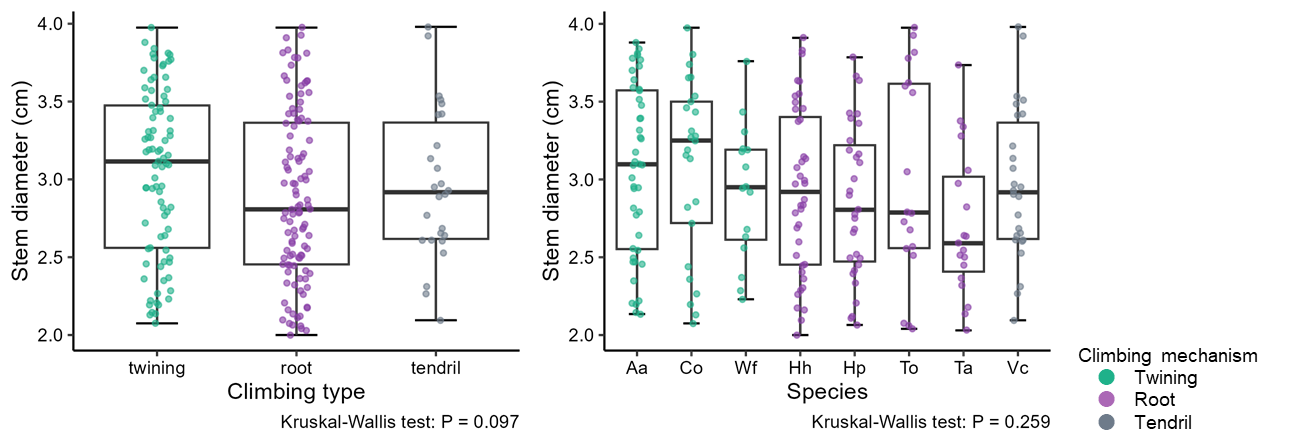


**Figure S5.** **Differences in stem diameter among climbing mechanisms and among liana species**

Points indicate individuals studied. Aa, *Actinidia arguta*; Co, *Celastrus orbiculatus*; Wf, *Wisteria floribunda*; Hh, *Hydrangea hydrangeoides*; Hp, *Hydrangea petiolaris*; To, *Toxicodendron orientale*; Ta, *Trachelospermum asiaticum*; Vc, *Vitis coignetiae*.


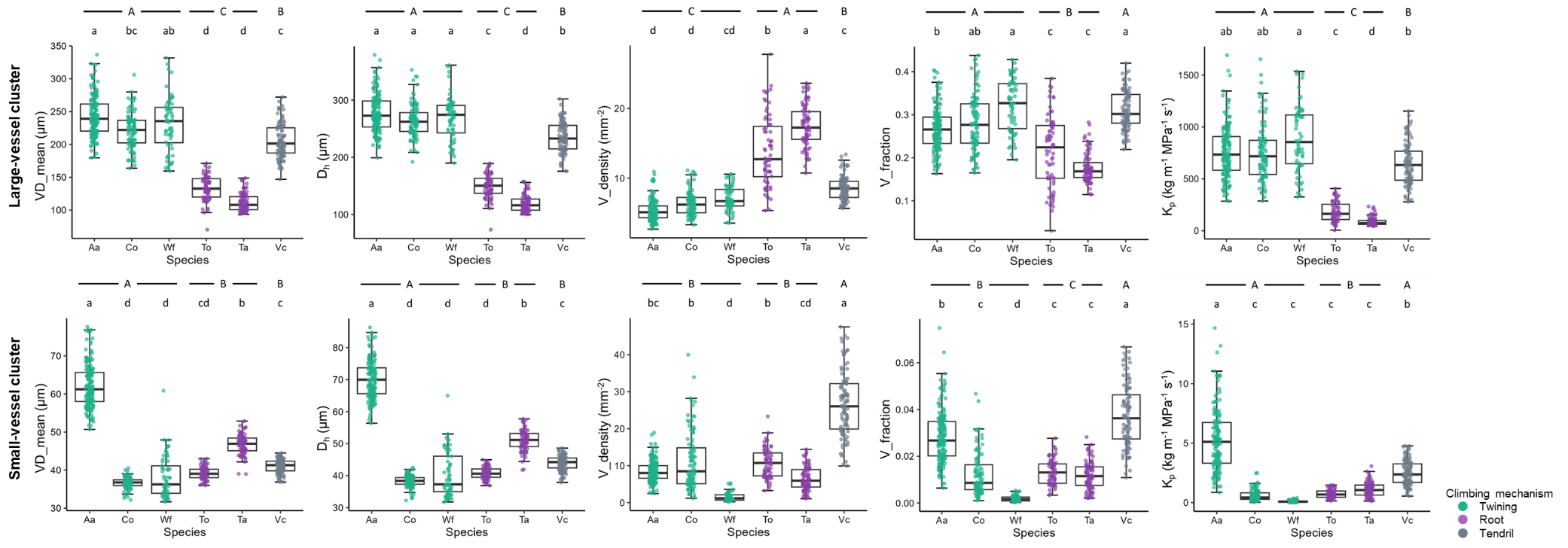


**Figure. S6.** **Differences in xylem vessel traits among climbing mechanisms and among species in each vessel cluster of lianas**

Each point represents the value for each transverse section. Upper panels indicate traits of large-vessel clusters and lower panels indicate traits of small-vessel clusters. Species not exhibiting vessel dimorphism (*Hydrangea hydrangeoides* and *Hydrangea petiolaris*) were excluded. Letters above each panel are assigned in descending order of mean trait values; different letters indicate significant differences in mean trait values among species or climbing mechanisms (Tukey’s honestly significant difference test, *P* < 0.05). Upper-case letters indicate differences among climbing mechanisms and lower-case letters indicate differences among species. VD_mean, mean vessel diameter; *D*_h_, mean hydraulic weighted vessel diameter; V_density, vessel density; V_fraction, vessel fraction; *K*_p_, potential hydraulic conductivity. Aa, *Actinidia arguta*; Co, *Celastrus orbiculatus*; Wf, *Wisteria floribunda*; Hh, *Hydrangea hydrangeoides*; Hp, *Hydrangea petiolaris*; To, *Toxicodendron orientale*; Ta, *Trachelospermum asiaticum*; Vc, *Vitis coignetiae*.

**
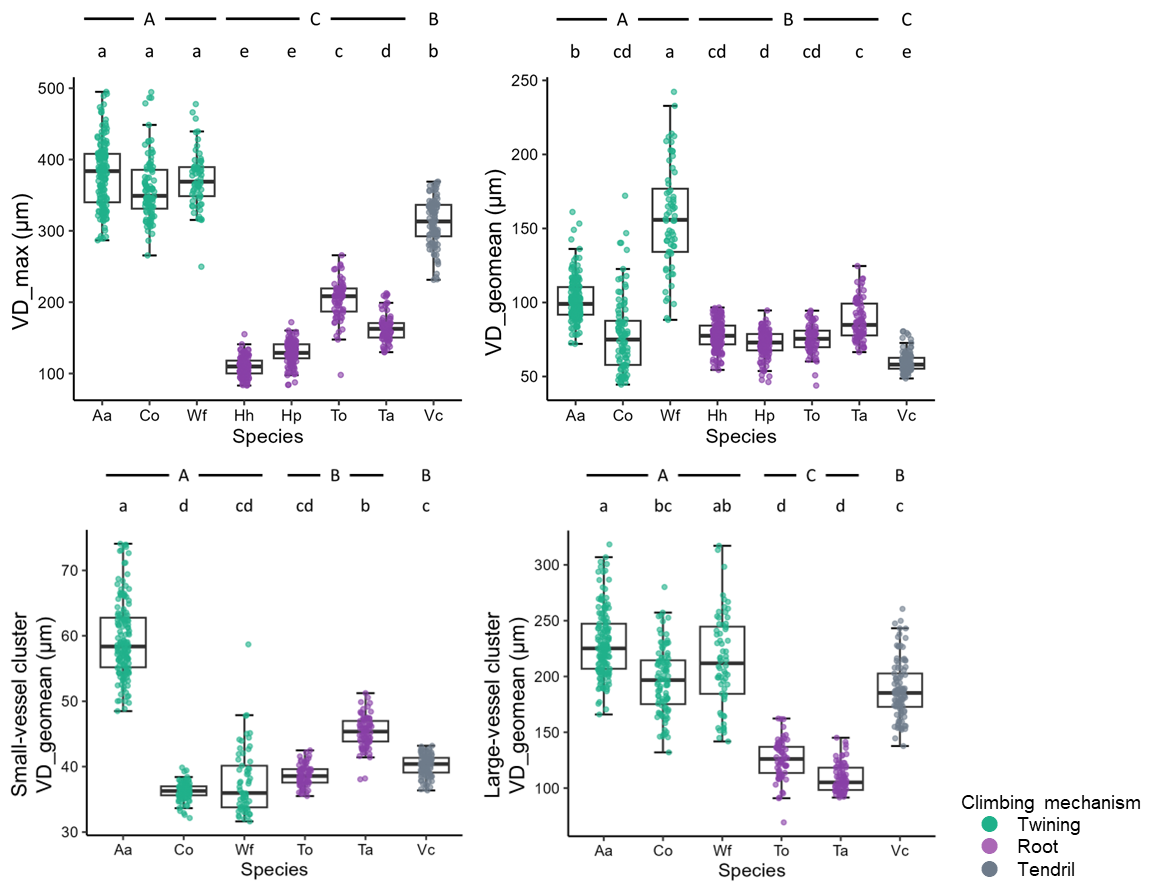
**

**Figure S7.** **Supplementary results for vessel trait comparisons among climbing mechanisms and among species of lianas**

Each point represents the value for each transverse section. Letters above each panel are assigned in descending order of mean trait values; different letters indicate significant differences in mean trait values among species or climbing mechanisms (Tukey’s honestly significant difference test, *P* < 0.05). Upper-case letters indicate differences among climbing mechanisms and lower-case letters indicate differences among species. Species not exhibiting vessel dimorphism (*Hydrangea hydrangeoides* and *Hydrangea petiolaris*) were excluded from the results for the small and large vessel clusters (bottom panels). VD_max, maximum vessel diameter; VD_geomean, geometric mean of vessel diameter; Aa, *Actinidia arguta*; Co, *Celastrus orbiculatus*; Wf, *Wisteria floribunda*; Hh, *H. hydrangeoides*; Hp, *H. petiolaris*; To, *Toxicodendron orientale*; Ta, *Trachelospermum asiaticum*; Vc, *Vitis coignetiae*.

**Supplementary information 2.**

**Supplementary Table.**

**Table S1.** Representative values of traits in liana species in the study

| **Species** | **Climbing mechanism** | **Leaf phenology** | **VD_mean** | **VD_max** | ***D*_h_** | **V_density** | **V_fraction** | ***K*_p_** |
| --- | --- | --- | --- | --- | --- | --- | --- | --- |
|  |  |  | **μm** | **μm** | **μm** | **mm^−2^** |  | **kg m^-1^ MPa^−1^ s^−1^** |
| *Actinidia arguta* (Siebold et Zucc.) Planch. ex Miq. | twining | DB | 133.72 (19.08) | 494.89 | 218.65 (26.39) | 13.82 (3.68) | 0.295 (0.050) | 766.08 (251.26) |
| *Celastrus orbiculatus* Thunb. var. *orbiculatus* | twining | DB | 114.36 (33.61) | 494.47 | 207.73 (21.68) | 17.45 (7.82) | 0.292 (0.067) | 754.96 (274.46) |
| *Wisteria floribunda* (Willd.) DC. | twining | DB | 195.92 (33.90) | 477.72 | 254.44 (34.08) | 8.56 (1.81) | 0.324 (0.060) | 886.64 (314.46) |
| *Hydrangea hydrangeoides* (Siebold et Zucc.) B. Schulz | root | DB | 79.33 (9.44) | 155.13 | 82.44 (9.38) | 80.84 (20.05) | 0.397 (0.049) | 89.91 (25.42) |
| *Hydrangea petiolaris* Siebold et Zucc. | root | DB | 76.76 (9.81) | 171.91 | 86.48 (11.18) | 73.93 (15.78) | 0.366 (0.052) | 102.04 (33.17) |
| *Toxicodendron orientale* Greene | root | DB | 92.33 (12.63) | 265.8 | 129.46 (17.64) | 24.58 (7.11) | 0.228 (0.085) | 183.83 (98.24) |
| *Trachelospermum asiaticum* (Siebold et Zucc.) Nakai | root | EB | 95.61 (14.48) | 212.54 | 110.97 (15.57) | 23.94 (5.25) | 0.189 (0.033) | 90.41 (41.66) |
| *Vitis coignetiae* Pulliat ex Planch. | tendril | DB | 82.52 (12.25) | 368.99 | 165.8 (18.58) | 35.24 (9.10) | 0.347 (0.044) | 644.57 (196.92) |

VD_mean, mean vessel diameter; VD_max, maximum vessel diameter; *D*_h_, mean hydraulically weighted vessel diameter; V_density, vessel density; V_fraction, vessel fraction; *K*_p_, potential hydraulic conductivity. Trait values indicate mean values for each species, with the exception of VD_max, which indicates the maximum value for the species. Values within parentheses are standard deviation.
